# Dynamic and stochastic systems as a framework for metaphysics and the philosophy of science

**DOI:** 10.1007/s11229-019-02231-8

**Published:** 2019-09-03

**Authors:** Christian List, Marcus Pivato

**Affiliations:** 1 Department of Philosophy, Logic, and Scientific Method, London School of Economics, London, UK; 2 THEMA, Université de Cergy-Pontoise, Cergy-Pontoise, France

**Keywords:** Dynamical systems, Stochastic processes, Nomological possibility and necessity, Determinism, Indeterminism, Laws, Symmetries, Regularities, Scientific inference, Ergodicity, Occam’s Razor, Space, Time, Formal metaphysics

## Abstract

Scientists often think of the world (or some part of it) as a dynamical system, a stochastic process, or a generalization of such a system. Prominent examples of systems are (i) the system of planets orbiting the sun or any other classical mechanical system, (ii) a hydrogen atom or any other quantum–mechanical system, and (iii) the earth’s atmosphere or any other statistical mechanical system. We introduce a general and unified framework for describing such systems and show how it can be used to examine some familiar philosophical questions, including the following: how can we define nomological possibility, necessity, determinism, and indeterminism; what are symmetries and laws; what regularities must a system display to make scientific inference possible; how might principles of parsimony such as Occam’s Razor help when we make such inferences; what is the role of space and time in a system; and might they be emergent features? Our framework is intended to serve as a toolbox for the formal analysis of systems that is applicable in several areas of philosophy.

## Introduction

For both scientific and philosophical purposes, we often find it useful to think of the world (or some part of it that we are studying) as a system evolving over time: a dynamical system, a stochastic process, or a suitable generalization of such a system. In both science and philosophy, many theories represent the world (or the part they are concerned with) in terms of such systems, with various structures and properties. Metaphysical commitments often take the form of claims about the nature of those structures and properties: which of them are real and not just artefacts of our models, which are fundamental as opposed to derivative, and which are necessary as opposed to contingent.

In this paper, we introduce a general and unified framework for describing systems, based on the theory of dynamical systems and stochastic processes, and show how this framework can be used to examine and illuminate some familiar philosophical questions. Here are some examples:

- What does it mean for a system to be deterministic or indeterministic, and which features of the system, if any, determine which others?
- Does the present determine the future? Does it determine the past? What is the smallest set of facts encoding the system’s entire history? Could there be non-temporal forms of determinism?
- How can we define nomological possibility and necessity for a system?
- What are the laws governing a particular system, and is there a distinction between “laws” and “brute necessities”? How do laws depend on symmetries?
- What structure must a system have in order to permit generalizations from local observations to global regularities?
- How might we use principles of parsimony such as Occam’s Razor when we make such generalizations? And can we formulate a version of Occam’s Razor in terms of symmetries?
- What is the role of space and time in a system? What is the relationship between the geometry of space and time and the system’s behaviour?
- Is this spatiotemporal geometry exogenous, or is it determined by the dynamics? In other words, are space and time more fundamental than the system’s dynamics, or the other way around? Might space and time be “emergent”?
- How should we individuate systems? Should two structurally indistinguishable systems count as “the same”, or might they count as different?

For each of these questions, our framework allows us to identify in clear and precise terms what is at stake. We illustrate the generality of the framework by sketching how it can accommodate, schematically, the systems described by some standard physical theories, such as classical mechanics, electrodynamics, quantum mechanics, and special and general relativity. In principle, our framework can also be used to describe many systems studied in the special sciences, such as biological, social, and economic systems, though we do not have the space to develop these applications here. We make a few remarks about special-science systems at the end of the paper and hope that our framework will serve as a basis for future work in some of those areas.^1^

The paper is structured as follows. We discuss three classes of systems, in increasing order of generality. We call the first *temporally evolving systems* (Sect. 2), the second *spatially extended systems* (Sect. 3), and the third *amorphous systems* (Sect. 4). We offer a conceptual toolbox for describing and analysing each class of systems, covering notions such as states and histories, determinism and indeterminism, nomological possibility and necessity, modal and probabilistic properties, symmetries and laws, ergodicity and its significance in making scientific inference possible, Occam’s Razor, and the role of time and/or space. We first explain all of these notions in the context of the simplest class of systems (in Sect. 2) and then generalize from there (in Sects. 3 and 4). The paper also includes some more technical appendices, on factor systems (relevant to the analysis of systems at different levels of abstraction), on partial and local symmetries (relevant to “local” laws and the analysis of systems with special initial or boundary conditions), on criteria of parsimony in relation to which symmetries to postulate (relevant to Occam’s Razor), and on the definition of spatial distance in quantum–mechanical systems (which raises special challenges).

Although the paper presupposes a willingness to engage with technical material—and a basic familiarity with science will be helpful—our goal is to keep the exposition as simple and self-contained as possible. Our intended contribution is twofold: methodological and substantive. On the methodological side, we aim to offer a unified and yet accessible framework for the philosophical analysis of many of the systems studied in the sciences. While the basic ideas originate from the theory of dynamical systems and stochastic processes in mathematics and physics, and partially overlapping formalisms can be found in earlier works (e.g., by Earman 1986; van Fraassen 1989; Frigg et al. 2011; Werndl 2009a, b; Bishop 2011; Butterfield 2012; Yoshimi 2012), the key ideas remain underappreciated in philosophy, and to our knowledge, an equally unified (and, we think, accessible) framework is not yet available in the philosophical literature.

On the substantive side, we aim to offer a number of novel insights, for example concerning (i) the nature of nomological possibility and necessity in a system and the definition of determinism and indeterminism, (ii) the role of symmetries in distinguishing between “laws” and “brute necessities” in a system, (iii) the significance of symmetries and ergodicity as prerequisites for scientific inference, (iv) the relationship between Occam’s Razor and the symmetries of a system, and (v) the possibility that the topology and geometry of space and time may be emergent properties resulting from a system’s correlation structure. These, we hope, will be useful substantive contributions, over and above the paper’s unificatory contribution.

## Temporally evolving systems

### Basic definitions

We begin with the simplest class of systems whose states evolve over time.^2^ To define a system in this class, we need to specify what *time* is, what the system’s *states* are, and how these states may *evolve* over time. *Time* is represented by a set of points *T* that is linearly ordered; we write < for the “before” relation. The *state* of the system at each point in time is given by an element of some *state space X*. For the moment, we make no assumptions about the internal structure of the states in *X*; they are uninterpreted primitives. A *history* of the system, capturing “state evolution”, is a path through the state space, represented by a function *h* from *T* into *X*. For each time *t* in *T*, *h*(*t*) is the system’s state at time *t*. In a physical system, each state might be a completely specified microphysical state in which the system could be at a particular point in time, and histories would be possible trajectories of the system through its state space over time.

We write Ω to denote the set of all histories deemed *possible*. Histories play the role of possible worlds. Thus, the structure of Ω reflects the notion of possibility we wish to capture. If we are interested in logical possibility, then Ω is simply the set of all logically possible functions from *T* into *X*, which we call ${\mathcal {H}}$. If we are interested in some form of nomological possibility, such as physical possibility, Ω will often be a proper subset of ${\mathcal {H}}$. Our intended interpretation of possibility throughout this paper is the *nomological* one, since we want to distinguish between histories that are permitted by the laws governing our system and histories that are not.

Subsets of Ω are called *events*. We can apply logical operations to events. The *conjunction* of two events *E* and $E^{\prime}$ is given by their intersection *E* ∩ *E′*. Their *disjunction* is given by their union *E* ∪ *E′*. The *negation* of an event *E* is given by its *complement* ~ *E* = Ω\*E*. Later we introduce *possibility* and *necessity* operators.

To complete the definition of a temporally evolving system, we must define probabilities on Ω. Formally, we introduce a *conditional probability structure*.^3^ This is a family of conditional probability functions {*Pr*_*E*_}_*E*⊆Ω_, consisting of one *Pr*_*E*_ for each event *E* in Ω, where *Pr*_*E*_ assigns to any event in Ω the conditional probability of that event, given *E*.^4^ The family must satisfy certain consistency conditions, such as compatibility with Bayesian conditionalization.^5^ Now, a *temporally evolving system* is the pair consisting of the set Ω of possible histories and the conditional probability structure {*Pr*_*E*_}_*E*⊆Ω_.

For example, in a weather system, *X* would be the set of all possible weather states and Ω the set of all possible weather histories. For each particular weather event *E*, say a hot temperature on Monday, the function *Pr*_*E*_ assigns to every weather event *D*, say a thunderstorm on Tuesday, the conditional probability of its occurrence, given *E*.

In principle, the probability structure admits two interpretations. Under an *objectivist* interpretation, it is a feature of the system itself and thus represents *objective chance* (see, e.g., Lewis 1986; Schaffer 2007; List and Pivato 2015). Of course, objective chance could be *degenerate*, i.e., restricted to the extremal values 0 or 1. Degenerate objective chance is a much-discussed feature of deterministic systems; we return to this point later. Under a *subjectivist* interpretation, the probability structure is not a feature of the system itself, but represents an observer’s beliefs about the system, as in subjective Bayesianism (e.g., de Finetti 1972). The most natural way to read this paper is to assume the objectivist interpretation, though our formalism itself is neutral.

Familiar examples of temporally evolving systems are the system of planets orbiting the sun or any other classical mechanical system, a hydrogen atom or any other quantum–mechanical system, the earth’s climate system or any other statistical mechanical system, and (arguably) the global economy or some other closed macro-economic system. Generally, any classical *dynamical system* is a special case of a temporally evolving system, as is any *stochastic process* under the standard definition.^6^

For theoretical simplicity, we focus on *closed* systems, which are not subject to any external perturbations. However, one could also represent *open* systems in our framework, by encoding any external perturbations as additional sources of randomness in the system’s conditional probability structure (“random forcings”).^7^

### Determinism and indeterminism

Conventionally, a system is called *deterministic* if, in that system, the past always determines the future. Formally, for any history *h* and any point in time *t*, let *h*_*t*_ be the *initial segment* of that history up to *t*. This is the function *h* restricted to the points in time up to *t*. History *h* is *deterministic* if, at any time *t* in *T*, the initial segment *h*_*t*_ admits only one possible continuation in Ω, where a *continuation* of *h*_*t*_ is a history *h′* such that $h^{{\prime }} _{t}$ = *h*_*t*_. History *h* is *indeterministic* if, for some time *t*, *h*_*t*_ has more than one possible continuation in Ω. The system as a whole is called *deterministic* if all histories in Ω are deterministic, and *indeterministic* if some histories in Ω are indeterministic.^8^

For example, classical mechanical systems, such as the solar system on the Newtonian picture, are deterministic. By contrast, quantum–mechanical systems, such as a decaying uranium atom, are indeterministic (assuming no hidden variables). If the wave function, which encodes the state of the quantum system, collapses at time *t*, the initial segment *h*_*t*_ of the system’s history *h* can admit multiple continuations.

Indeterministic systems allow non-degenerate chance as we move along a given history, while deterministic systems do not.^9^ To see this, note that the chance of any event *E* in history *h* at time *t* is the conditional probability of *E*, given that the initial segment *h*_*t*_ has occurred. Since the event that the initial segment *h*_*t*_ has occurred is given by the set of all continuations of *h*_*t*_—call this set [*h*_*t*_]—the probability in question is $Pr_{{[h_{t} ]}}$(*E*). If history *h* is deterministic, the entire conditional probability function $Pr_{{[h_{t} ]}}$ is *degenerate*, i.e., it assigns probability 0 or 1 to every event *E*. This is because the initial segment *h*_*t*_ has only one continuation, namely *h* itself, and so the specified event [*h*_*t*_] contains only a single history, *h*. Then $Pr_{{[h_{t} ]}}$(*E*) is 1 if *h* belongs to *E* and 0 otherwise. In contrast, if history *h* is indeterministic, $Pr_{{[h_{t} ]}}$ may be *non*-*degenerate*, assigning probabilities strictly between 0 and 1 to some events *E*. This is because [*h*_*t*_] need not be singleton here, and so $Pr_{{[h_{t} ]}}$ is less constrained. (For the moment, we set aside phenomena such as “higher-level” indeterminism and chance, as discussed in List and Pivato 2015. We briefly consider such phenomena at the end of this paper.)

Our framework also allows us to formulate some more general, less familiar notions of determinism. For *any* subset *T′* of *T*—not just the set of time points up to a particular time *t*—we can ask whether the restriction of a given history to the points in *T′* uniquely determines the rest of that history. Let *h*_*T′*_ denote the restriction of the function *h* to *T′*. Our question, then, is whether *h*_*T′*_ has a unique extension to all of *T* in Ω, where an *extension* of *h*_*T′*_ is a history *h′* such that $h^{{\prime }} _{{T^{{\prime }} }}  = h_{{T^{{\prime }} }}$. If there is a unique extension, history *h* may be called *T′*-*deterministic*.^10^

We might ask, for instance, whether the entire history of a system, both past and future, is determined by its present state alone. Similarly, we might ask whether, given the states of the system at two points in time, there is a unique history connecting them. So, one can in principle consider not only the familiar idea of “past-to-future” determinism, but also other forms of “local-to-global” determinism. In Sect. 3.2, we develop these ideas further and consider, among other things, spatial rather than temporal forms of determinism as well as locally restricted ones.

### Nomological possibility and necessity

We can explicitly define the notions of nomological necessity and possibility in our framework.^11^ Intuitively, an event *E* is nomologically possible in history *h* at time *t* if the initial segment of that history up to *t* admits *at least one* continuation in Ω that lies in *E*; and *E* is nomologically necessary in *h* at *t* if *every* continuation of the history’s initial segment up to *t* lies in *E*.

More formally, we say that one history, *h′*, is *accessible* from another, *h*, at time *t* if the initial segments of *h* and *h′* up to time *t* coincide, i.e., $h_{t} = h^{{\prime }}_{t}$. We then write *hR*_*t*_*h′*. The binary relation *R*_*t*_ on possible histories is in fact an equivalence relation (reflexive, symmetric, and transitive). Now, an event *E* ⊆ Ω is *nomologically possible* in history *h* at time *t* if *some* history *h′* in Ω that is accessible from *h* at *t* is contained in *E*. Similarly, an event *E* ⊆ Ω is *nomologically necessary* in history *h* at time *t* if *every* history *h′* in Ω that is accessible from *h* at *t* is contained in *E*.

We can thus define two modal operators, ◆_*t*_ and ■_*t*_, to represent possibility and necessity at time *t*. We define each of them as a mapping from events to events. For any event *E* ⊆ Ω,

- ◆_*t*_ *E* = {*h* ∈ Ω: for some *h′* ∈ Ω with *hR*_*t*_*h′*, we have *h′* ∈ *E*},
- ■_*t*_ *E* = {*h* ∈ Ω: for all *h′* ∈ Ω with *hR*_*t*_*h′*, we have *h′* ∈ *E*}.

So, ◆_*t*_ *E* is the set of all histories in which *E* is possible at time *t*, and ■_*t*_ *E* is the set of all histories in which *E* is necessary at time *t*. Accordingly, we say that ◆_*t*_ *E* holds in history *h* if *h* is an element of ◆_*t*_ *E*, and ■_*t*_ *E* holds in *h* if *h* is an element of ■_*t*_ *E*. As one would expect, the two modal operators are *duals* of each other: for any event *E* ⊆ Ω, we have ■_*t*_ *E* = ~ ◆_*t*_ ~ *E* and ◆_*t*_ *E* = ~ ■_*t*_ ~ *E*.

Two remarks are due. First, although we have here defined *nomological* possibility and necessity, we can analogously define *logical* possibility and necessity. To do this, we must simply replace every occurrence of the set Ω of nomologically possible histories in our definitions with the set $\cal{H}$ of logically possible histories. Second, by defining the operators ◆_*t*_ and ■_*t*_ as functions from events to events, we have adopted a *semantic* definition of these modal notions. However, one could also describe them *syntactically*, by introducing an explicit modal logic. For each point in time *t*, the logic corresponding to the operators ◆_*t*_ and ■_*t*_ would then be an instance of a standard S5 modal logic (on S5, see, e.g., Priest 2001).

Our analysis shows how nomological possibility and necessity depend on the dynamics of the system, as evident from the time-indexed nature of the relevant modal operators. In particular, as time progresses, the notion of possibility becomes more demanding: fewer events remain possible at each time. And the notion of necessity becomes less demanding: more events become necessary at each time, for instance due to having been “settled” in the past. Formally, for any *t* and *t′* in *T* with *t* < *t′* and any event *E* ⊆ Ω,

- if ◆_*t′*_ *E* then ◆_*t*_ *E*,
- if ■_*t*_ *E* then ■_*t′*_ *E*.

Furthermore, in a deterministic system, for every event *E* and any time *t*, we have ◆_*t*_ *E* = ■_*t*_ *E*. In other words, an event is possible in any history *h* at time *t* if and only if it is necessary in *h* at *t*. In an indeterministic system, by contrast, necessity and possibility come apart.

Just as we previously discussed different notions of determinism—not just “past to future” but also “local to global”—so we can generalize the notions of possibility and necessity in a similar way. Let us say that one history, *h′*, is *accessible* from another, *h*, relative to a set *T′* of time points, if the restrictions of *h* and *h′* to *T′* coincide, i.e., *h′*_*T′*_ = *h*_*T′*_. We then write *hR*_*T′*_*h′*. Accessibility at time *t* is the special case where *T′* is the set of points in time up to time *t*. We can define nomological possibility and necessity relative to *T′* as follows. For any event *E* ⊆ Ω,

- ◆_*T′*_ *E* = {*h* ∈ Ω: for some *h′* ∈ Ω with *hR*_*T′*_*h′*, we have *h′* ∈ *E*},
- ■_*T′*_ *E* = {*h* ∈ Ω: for all *h′* ∈ Ω with *hR*_*T′*_*h′*, we have *h′* ∈ *E*}.

Although these modal notions are much less familiar than the standard ones (possibility and necessity at time *t*), they are useful for some purposes. In particular, they allow us to express the fact that the states of a system during a particular period of time, *T′* ⊆ *T*, render some events *E* possible or necessary.

Finally, our definitions of possibility and necessity relative to some general subset *T′* of *T* allow us to define completely “atemporal” notions of possibility and necessity. If we take *T′* to be the empty set, then the accessibility relation *R*_*T′*_ becomes the universal relation, under which every history is related to every other. An event *E* is possible in this atemporal sense (i.e., ◆_∅_*E*) if and only if *E* is a non-empty subset of Ω, and it is necessary in this atemporal sense (i.e., ■_∅_*E*) if *E* coincides with all of Ω. These notions might be viewed as possibility and necessity from the perspective of some observer who has no temporal or historical location within the system and looks at it from the outside.

### Modal and probabilistic properties

Ultimately, all modal properties of a temporally evolving system are encoded by the set Ω of nomologically possible histories, and all probabilistic properties are encoded by the conditional probability structure {*Pr*_*E*_}_*E*⊆Ω_. This raises the question: which, if any, of these properties qualify as “laws” of the system, and what does this mean?

One possible view is that:

- *any* property that is satisfied by *all* histories in Ω counts as a law of the system, specifically a “modal law”; and
- *any* property of the conditional probability structure {*Pr*_*E*_}_*E*⊆Ω_ counts as a law of the system, specifically a “probabilistic law”.

Indeed, since the system is fully specified by Ω and {*Pr*_*E*_}_*E*⊆Ω_, one might interpret anything that is globally true of its possible histories or its probability structure as a law of that system. A view along these lines is expressed in a classic paper by Sellars (1948, p. 309): “A natural law is a universal proposition, implicative in form, which holds of all histories of a family of possible histories; as such it is distinguished from ‘accidental’ formal implications which hold of one or more possible histories of the family, but do not hold of all.” So, the notions “being a law” and “being nomologically necessary” essentially coincide.

Against this view, however, we want to argue that even among nomologically necessary properties of a system—those that are not contingent on particular histories—one can distinguish between “laws” on the one hand and “brute necessities”, which are not law-like, on the other. Laws, we suggest, have a testable and generalizable character which brute necessities lack. To explain this, we introduce two preliminary notions, *properties of histories* and *probabilistic properties*, and then provide a criterion for identifying which of them qualify as laws.

A *property of histories*, *P*, is a binary feature that a history may or may not have. Formally, it can be associated with some subset, denoted [*P*], of the set ${\mathcal {H}}$ of all logically possible histories. A history *h* satisfies *P* if *h* belongs to [*P*]. We call [*P*] the *extension* of *P*. A property satisfied by *every* history in Ω can be called *nomologically necessary* for the system. Newton’s three laws of motion are examples of such properties in the case of a classical mechanical system.

A *probabilistic property*, ${\mathcal {P}}$, is a binary feature that a conditional probability structure may or may not have. Formally, it is associated with a subset, denoted [${\mathcal {P}}$], of the set Π of all logically possible conditional probability structures on Ω. A conditional probability structure {*Pr*_*E*_}_*E*⊆Ω_ satisfies ${\mathcal {P}}$ if it belongs to [${\mathcal {P}}$]. We call [${\mathcal {P}}$] the *extension* of ${\mathcal {P}}$. An example of a probabilistic property is the one that says: “The unconditional probability of event *F* is ½.” Its extension is the set of all conditional probability structures {*Pr*_*E*_}_*E*⊆Ω_ for which *Pr*_Ω_(*F*) = ½. Another example is the second law of thermodynamics. This is a probabilistic property that is satisfied by the conditional probability structure of a statistical mechanical system.

Our goal is to distinguish between those properties that qualify as “laws” of the system and those that do not. We capture that distinction through the notion of *symmetries*. Informally, a *symmetry* is a transformation that acts on either the state space *X* or the set of time points *T*, or both, and which can capture certain admissible changes in perspective on the system. *Laws*, we suggest, are those nomologically necessary properties which are invariant under symmetries and which therefore hold across changes in perspective. We now make this formally precise.

### Symmetries

We first consider symmetries acting on the state space; we then turn to symmetries acting on time; and we finally consider more general symmetries. To introduce *state symmetries*, we begin with some preliminary definitions. Let ϕ be any function from *X* into itself, i.e., a transformation on the state space. We use this transformation to define a function from histories to other histories. For reasons that will become clear, we do not restrict the function to nomologically possible histories, but define it as a function on ${\mathcal {H}}$, the set of all logically possible histories. Specifically, for any history *h* in ${\mathcal {H}}$, we define the transformed history

$$ \upphi(h) = h^{{\prime }} ,\;{\text{where,}}\;{\text{for}}\;{\text{all}}\;t\;{\text{in}}\,T,h^{{\prime }} (t) =\upphi(h(t)). $$

For example, if *X* = {*a*, *b*, *c*, *d*,*…*, *z*}, the function ϕ might shift every letter in the alphabet one place to the right, i.e., *a* to *b*, *b* to *c*, and so on, and *z* back to *a*. If we represent histories as sequences of elements in *X*, interpreted as the system’s states at times 1, 2, 3, …, then applying ϕ to the history *h* = (*b*, *a*, *c*, *f*, *z*,…) yields the history *h′* = (*c*, *b*, *d*, *g*, *a*,…). For convenience, we use the letter ϕ to denote both the original function on the state space and the induced function on the set ${\mathcal {H}}$ of histories. Note that since the set of nomologically possible histories may be a proper subset of the set of all logically possible histories, the image of a history in Ω need not be in Ω.

To define what it means for ϕ to be a symmetry, we need one further preliminary definition. For any collection of histories *E* in ${\mathcal {H}}$, the *inverse image* of *E* under ϕ is the set of all histories *h* in ${\mathcal {H}}$ such that ϕ(*h*) lies in *E*.^12^ For example, if *E* is the set of all histories whose state at time 3 is *c*, and ϕ is the letter-shifting transformation, then the inverse image of *E* under ϕ is the set of all histories whose state at time 3 is *b*. Now, the function ϕ is a *symmetry* of our system if

- ϕ(Ω) = Ω, i.e., (i) ϕ(*h*) is in Ω, for all *h* in Ω, and (ii) for any *h* in Ω, there is some *h′* in Ω such that ϕ(*h′*) = *h*; and
- for any events *E* and *D* in Ω, if *E′* and *D′* are the inverse images of *E* and *D* under ϕ, then *Pr*_*E′*_(*D′*) = *Pr*_*E*_(*D*).^13^

Intuitively, a symmetry is a transformation that preserves the system’s modal and probabilistic structure. In our example, where *X* = {*a*, *b*, *c*, *d*,*…*, *z*} and ϕ is the letter-shifting function, the first part of this definition implies that the set of nomologically possible histories is preserved under shifting of letters. For instance, if (*b*, *a*, *c*, *f*, *z*,…) is a nomologically possible history, then so is (*c*, *b*, *d*, *g*, *a*,…).^14^ To illustrate the second part, let *E* be the set of all histories in Ω whose state at time 3 is *c*, and let *D* be the set of all histories in Ω whose state at time 5 is *a* (so that *E′* is a suitable set of histories whose state at time 3 is *b*, and *D′* is a suitable set of histories whose state at time 5 is *z*).^15^ The conditional probability that the state of a history at time 5 is *a*, given that at time 3 it is *c*, must then equal the conditional probability that the state at time 5 is *z*, given that at time 3 it is *b*.

Obviously, not all state transformations are symmetries. Whether there are any non-trivial state symmetries depends on the system in question, i.e., it depends on Ω and {*Pr*_*E*_}_*E*⊆Ω_. In classical mechanical systems, state symmetries include spatial translations, which shift everything in a certain direction by a certain distance, rotations and reflections, and permutations of particles with equal mass. Those transformations preserve the modal and probabilistic structure of the relevant systems.

Similarly, we can define *time symmetries*. Again, we begin with some preliminary definitions. Let ψ be any function on *T*, i.e., a transformation on time. For any history *h*, we define the transformed history^16^

$$ \uppsi(h) = h^{{\prime }} ,\;{\text{where,}}\;{\text{for}}\;{\text{all}}\;t\;{\text{in}}\;T,h^{{\prime }} (t) = h(\uppsi(t)). $$

For example, if *T* = {1,2,3,…}, the function ψ might be given by ψ(*t*) = *t* + 5 for all *t* in *T*. It maps the history (*x*_1_, *x*_2_, *x*_3_, …) (a sequence of states across time) to the history (*x*_6_, *x*_7_, *x*_8_, …). As in the case of state symmetries, ψ induces a function from the set ${\mathcal {H}}$ to itself. In analogy to the earlier definition, ψ is a *symmetry* if

- ψ(Ω) = Ω;
- for any events *E* and *D* in Ω, if *E′* and *D′* are the inverse images of *E* and *D* under ψ, then *Pr*_*E′*_ (*D′*) = *Pr*_*E*_(*D*).

In our example, where *T* = {1,2,3,…} and ψ(*t*) = *t* + 5, the first part of this definition implies that if *h* = (*x*_1_, *x*_2_, *x*_3_,…) is a nomologically possible history of the system, then so is *h′* = (*x*_6_, *x*_7_, *x*_8_,…).^17^ To illustrate the second part, suppose that *E* is the set of all histories in Ω whose state at time 3 is *c*, while *D* is the set of all histories in Ω whose state at time 4 is *a* (so that *E′* is a suitable set of histories whose state at time 8 is *c*, while *D′* is a suitable set of histories whose state at time 9 is *a*). The conditional probability that the state at time 9 is *a*, given that at time 8 it was *c*, must then equal the conditional probability that the state at time 4 is *a*, given that at time 3 it was *c*.^18^

Just as not all state transformations are symmetries, so not all time transformations are symmetries either. In most classical physical systems, time symmetries include *time translations*, such as ψ(*t*) = *t* + 5, but exclude *non*-*linear transformations*, such as ψ(*t*) = *t*^2^. In systems where the state does not encode explicitly “kinetic” properties (such as momentum), *simple time reversals*, such as ψ(*t*) =  − *t*, can also be time symmetries. For example, the partial differential equations describing wave propagation in an ideal medium are invariant under simple time reversals. But many other systems, such as thermodynamic ones and diffusion processes, do not admit such simple time reversals.

More general symmetries include composite functions resulting from the combination of transformations of *X* and transformations of *T*. These are best understood as functions acting on the set ${\mathcal {H}}$ of logically possible histories directly, with the properties introduced above. A familiar example in classical mechanical systems is a *time reversal*, which involves *both* a negation of the time index *and* a negation of all momentum vectors in the system (not to be confused with a *simple* *time reversal*, as mentioned earlier).^19^ A more complex example is a *Galilean transformation*, which adds a constant vector to the momentum vectors of all particles and also a time-varying sequence of spatial shifts to the particle positions, thereby converting the system to a different inertial reference frame. See footnote 48 below for details.

We can think of symmetries—whether they act on the state space, on time, or on both—as transformations that encode admissible changes in perspective on a system, insofar as they preserve the system’s modal and probabilistic structure. We write Ψ to denote the set of *all* symmetries of our temporally evolving system. This set has the algebraic structure of a *monoid*. Formally, a *monoid* is a set of transformations (here of ${\mathcal {H}}$) which (i) contains the *identity transformation* (mapping every history to itself) and (ii) is *closed under composition* (i.e., for any two transformations in the set, the transformation obtained by applying first one of the two transformations and then the other is also in the set). An example of a symmetry monoid is the set of all rotations of a classical mechanical system around a fixed axis: the identity transformation obviously belongs to this set, being a rotation by an angle of zero, and the composition of any two rotations is still a rotation.^20^

### Laws and their significance

As anticipated, the laws of a system are those nomologically necessary properties within it that are invariant under symmetries. This, we show, makes laws open to testing and generalization. Laws, one might say, have a “scrutable” and “projectable” character. The close relationship between symmetries and laws has been recognized before.^21^ For instance, Wigner (1967) takes symmetries to be “a prerequisite for the very possibility of discovering the laws of nature” (as Brading and Castellani 2013 put it; see also French 2014). And van Fraassen (1989), in his classic study of symmetries in science, considers defining laws as “facts which are invariant under symmetries”, though ultimately does not endorse that definition. But none of the existing accounts clarifies the relationship between laws and symmetries in a way that we consider fully satisfactory.^22^ We develop this relationship in detail in the case of modal laws. We subsequently consider probabilistic laws too, but, due to space constraints, discuss those more briefly.

To define the notion of a *modal law*, consider a property of histories, *P*. Recall that *P* is *nomologically necessary* for the given system if its extension, [*P*], includes all histories in Ω. For any symmetry ψ, we say that *P* is *invariant* under ψ if the set [*P*] is equal to its inverse image under ψ. Property *P* is a *law* if it is nomologically necessary for the system *and* invariant under all symmetries in Ψ.

For example, suppose *T* = {1, 2, 3,…}, and suppose that, for any non-negative integer *r*, the system has the time symmetry ψ_*r*_ defined by ψ_*r*_(*t*) = *t* + *r* for all *t* in *T*; for simplicity, the system has no other symmetries. So, Ψ = {ψ_*r*_: *r* = 0, 1, 2,…}. Now, suppose all histories in Ω satisfy property *P* which says: “If the state at time 5 is *x*, then at time 6 it is *y*.” Despite being nomologically necessary for the present system, this property falls short of being a law. The inverse image of [*P*] under any symmetry ψ_*r*_ corresponds to the property *P′* which says: “If the state of the system at time 5 + *r* is *x*, then at time 6 + *r* it is *y*.” Clearly, unless *r* = 0, [*P′*] is not the same as [*P*], and so *P* is *not* invariant under the system’s symmetries. We call such a property—nomologically necessary but not invariant under symmetries—a *brute necessity*.

By contrast, suppose all histories in Ω have the property *P* which says: “For any *t* in *T*, if the state of the system at time *t* is *x*, then at time *t* + 1 it is *y*.” It is easy to see that this property *is* invariant under all symmetries of the system: the inverse image of [*P*] under any symmetry ψ_*r*_ is the same as [*P*]. Thus, *P* is a law.

For another example, consider the kinds of temporally evolving systems that arise in classical mechanics. These satisfy the law of *conservation of energy*, which says that the total energy (kinetic plus potential) remains constant over time. This can be formulated as a property *P* of the form: “For any times *t* and *t′* in *T*, the total energy of the state at time *t′* equals the total energy of the state at time *t*.” Clearly, this property is invariant under the time symmetries {ψ_*r*_} introduced above. As already mentioned, classical mechanical systems also have certain *state* symmetries, such as spatial translations, rotations, reflections, and the permutation of (equal-mass) particles. The total energy of a state is unchanged by such symmetries too, so the property *P* will also be invariant under spatial translations and (equal-mass) particle permutations. Indeed, total energy is unchanged by every symmetry of the system, and for this reason, property *P* is a law.^23^

As we will now see, laws are testable and generalizable in a way in which properties that fall short of being laws are not, even if they are nomologically necessary. Suppose we are trying to figure out the status of some property *P*. Is it nomologically necessary? Does it capture a general regularity of our system? Is it a law? The first thing to note is that when we investigate a system, we are seldom able to observe all its nomologically possible histories. Conducting many “runs” of the same experiment is an attempt to observe as many histories as possible, but even the best experimental design rarely allows us to observe *all* possible histories. Furthermore, this strategy works only for smaller systems that we can isolate in laboratory conditions. When the system is the economy, the global ecosystem, or the universe as a whole, we are stuck in a single history. We cannot step outside that history and look at alternative histories. The observed history is the only evidence we have. Can we still say anything useful about the status of property *P*? It is at this point that symmetries come into play.

Let us return to our simply example of a system with *T* = {1, 2, 3,…} and time symmetries of the form ψ_*r*_, where ψ_*r*_(*t*) = *t*+*r*. Consider again the property *P* that says “if the state at time 5 is *x*, then at time 6 it is *y*”, and suppose, as before, that *P* is nomologically necessary, i.e., every history in Ω satisfies *P*. If we could observe many nomologically possible histories of the system, we would be able to verify the satisfaction of *P* in each case. But, as noted, we may be trapped in a single history, *h*. All we can do is watch this history unfold. We first see *h*(1), then *h*(2), then *h*(3), and so on. Importantly, we get to observe *h*(5) and *h*(6) only *once*, so we get only one chance to observe whether *h* satisfies property *P*. Furthermore, even if *h* does satisfy *P*, this is only a single data point, which tells us very little about the broader status of *P*. Property *P* might as well be a contingent feature of the actual history we have observed.

However, we *do* get to observe *h*(7), *h*(8), *h*(9), and so on. So, we *can* consider properties such as *P′*: “if the state at time 7 is *x*, then at time 8 it is *y*”; and *P″*: “if the state at time 9 is *x*, then at time 10 it is *y*”; and so on. Note that *P′* corresponds to the inverse image of [*P′*] under ψ_2_; and *P′* corresponds to the inverse image of [*P*] under ψ_4_; and so forth. In other words, if we are patient, we *can* observe whether history *h* satisfies the properties corresponding to the inverse images of the original property under a lot of elements of the system’s symmetry monoid. Similarly, in a system with spatial symmetries (of the sort we introduce in Sect. 3.5), we can in principle observe whether *h* satisfies the properties corresponding to many of the relevant inverse images simply by traveling in space.

Now, if property *P* was not itself invariant under symmetries, as in the case of our example, we would not learn much from this exercise. We would learn that *h* satisfies *P* (“if *h*(5) = *x*, then *h*(6) = *y*”), that it satisfies *P′* (“if *h*(7) = *x*, then *h*(8) = *y*”), and that it satisfies *P″* (“if *h*(9) = *x*, then *h*(10) = *y*”), and so on. But, strictly speaking, these are distinct properties, and on the face of it, they do not have all that much in common. By contrast, if *P* is symmetry-invariant, as in the case of the property which says “for all *t*, if *h*(*t*) = *x*, then *h*(*t* + 1) = *y*”, then *P*, *P′*, *P″*, … are all the *same* property, and thus the present exercise yields a whole series of experimental tests of the *same* law.

Moreover, in this case, the single property *P* picks up a general pattern, of which we can observe many instances even within a single history, *h*, and which lends itself to extrapolation into the future. As *h* unfolds, we can observe that state *x* is followed by state *y* not just once but many times. Furthermore, once we have observed this regularity a sufficient number of times, we may feel confident in hypothesizing that *P* is indeed a law and then predicting that, in the future, state *x* will also be followed by state *y*.

Contrast this with the case of a property that is not symmetry-invariant, such as “if *h*(5) = *x*, then *h*(6) = *y*”. Here, there is no such general pattern, and we have no basis for making any predictions. This is the sense in which laws have a *testable* and *generalizable* character that non-symmetry-invariant properties lack, even when they are nomologically necessary.

There is another way of making the same points. Let *P* be some property, and let *P′*, *P″*, *P‴*, and so on, be all of its inverse images under the various time (and other) symmetries of the system. Let *h* be the history that we observe. Suppose that, by exhaustive testing, we verify that *h* satisfies *P*, *P′*, *P″*, *P‴*, and so on. (Or perhaps we only verify some *subcollection* of these properties, but then infer the rest of them through some form of “empirical induction”, which is ubiquitous in science.) At this point, we have actually verified that *h* satisfies an entire conjunction of properties, informally *P* ∧ *P′* ∧ *P″* ∧ *P‴* ∧ …, or more formally, the property P* with extension

$$ [P^{*} ] = \bigcap\limits_{{\uppsi  \in \Psi }} {\uppsi ^{{ - 1}} ([P])} .$$

Note that, by construction, property P* is invariant under all symmetries in Ψ. Thus, although we get to test the initial property *P* only once, by testing a bunch of “*P*-like” properties at various points in time (and/or positions in space etc.), we have tested not only *P*, but something much *stronger*, namely P*. But note that P* is not just any arbitrary property: it is symmetry-invariant by construction and thus qualifies as a law (provided it is also nomologically necessary). Moreover, by entailing all the various instances of *P*-like properties, i.e., *P*, *P′*, *P″*, *P‴*, and so on, the hypothesis that property P* is a law allows us to make predictions as to what will happen at different points in time (or in space, or after making other admissible changes corresponding to symmetries of the system).

This argument suggests that *any* property that we think we have corroborated by performing a large number of empirical tests at different times (or locations in space, or different orientations of the experimental apparatus, or different collections of otherwise identical atoms, and so on) is *ipso facto* a symmetry-invariant law, and not merely a brute necessity.^24^

One can give a similar account of probabilistic laws. Let {$Pr^{\prime}_{E}$}_*E*⊆Ω_ be *any* conditional probability structure, and let ψ be a symmetry of the system. We define ψ({$Pr^{\prime}_{E}$}_*E*⊆Ω_) to be the conditional probability structure {$Pr^{\prime\prime}_{E}$}_*E*⊆Ω_ such that, for any events *E* and *D*, we have $Pr^{\prime\prime}_{E} (D) = Pr^{\prime}_{E^{\prime}} (D^{\prime})$, where *E′* and *D′* are, respectively, the inverse images of *E* and *D* under ψ. Let ${\mathcal {P}}$ be a probabilistic property. Recall that its extension, [${\mathcal {P}}$], is a subset of the set Π of all possible conditional probability structures on Ω. We say that ${\mathcal {P}}$ is *invariant* under ψ if [${\mathcal {P}}$] is equal to its inverse image under ψ. A property ${\mathcal {P}}$ that is satisfied by the system’s conditional probability structure {*Pr*_*E*_}_*Ε*⊆Ω_ is a *law* of the system if it is invariant under *all* symmetries in Ψ.

For example, suppose *T* = {1, 2, 3,…}, and let the time symmetries ψ_*r*_ be as defined before. Let *Y* and *Z* be two subsets of the state space *X*, and suppose the system’s conditional probability structure satisfies the probabilistic property ${\mathcal {P}}$ which says: “Conditional on the state being in *Y* at time 5, there is a 50% probability that the state will be in *Z* at time 6.” The inverse image of [${\mathcal {P}}$] under ψ_2_ corresponds to the property ${\mathcal {P}}^{\prime}$ which says: “Conditional on the state being in *Y* at time 7, there is a 50% probability that the state will be in *Z* at time 8.” Clearly, [${\mathcal {P}}^{\prime}$] is not the same as [${\mathcal {P}}$]. Thus, [${\mathcal {P}}$] is *not* invariant under ψ_2_, and so ${\mathcal {P}}$ is not a probabilistic law of the system.

However, suppose the conditional probability structure satisfies the property ${\mathcal {P}}$ which says: “For any time *t* in *T*, conditional on the state being in *Y* at time *t*, there is a 50% probability that the state will be in *Z* at time *t* + 1.” Then it is easy to see that [${\mathcal {P}}$] *is* invariant under ψ_*r*_ for all positive integers *r*. If Ψ consists only of the time symmetries {ψ_*r*_: *r* = 0, 1, 2, 3,…}, then ${\mathcal {P}}$ is invariant under *all* elements of Ψ, and so ${\mathcal {P}}$ is a probabilistic law.

As in the case of modal laws, probabilistic laws capture general and repeatable patterns. Consider again the probabilistic property ${\mathcal {P}}$ which says: “Conditional on the state being in *Y* at time 5, there is a 50% probability that the state will be in *Z* at time 6.” Recall that this property is not invariant under our system’s time symmetries. Even if the system’s conditional probability structure satisfies this property, the property does not capture a general pattern. It concerns only the probabilistic transition from time 5 to time 6. If, however, the system has all the time symmetries in Ψ, then we can expect the system to satisfy the properties corresponding to the inverse images of [${\mathcal {P}}$] under the various time symmetries, for instance: ${\mathcal {P}}^{\prime}$, which says: “conditional on the state being in *Y* at time 7, there is a 50% probability that the state will be in *Z* at time 8”; and ${\mathcal {P}}^{\prime\prime}$, which says: “conditional on the state being in *Y* at time 9, there is a 50% probability that the state will be in *Z* at time 10”; and so forth. By conjoining those properties, we can deduce the more general property ${\mathcal {P}}^{*}$, which says: “For any *t* in *T*, if the state of the system is in the set *Y* at time *t*, there is a 50% probability that it will be in *Z* at time *t* + 1.” This property *is* invariant under all the time symmetries, and it does indeed qualify as a law.

The foregoing considerations show that symmetries are central to the testable and generalizable character of laws. Without suitable symmetries, generalizing from local observations to global laws or testing hypothesized laws would not be possible, especially if we can observe only a single history of a given system. Nor would it be possible to make predictions about the future based on regularities observed in the past. In a slogan, for scientific inference and prediction to work, the system must have sufficient symmetries. In effect, when we engage in scientific reasoning about some system, or even about the world at large, we rely on the auxiliary hypothesis that this system, or the world, is sufficiently symmetrical. If our system, or the world, were what Cartwright (1999) calls “dappled”, then presumably we would not be able to presuppose such symmetries, and our ability to make scientific generalizations would be compromised.^25^

In “Appendix A”, we extend the present analysis to *factor systems*, which are obtained by abstracting away from certain details of the original system. In “Appendix B”, we extend it to *partial* and *local* symmetries, which are often found in systems with special initial conditions and/or boundary conditions.^26^

### Ergodicity and its significance

We have noted that, when we scientifically investigate a system, we rely heavily on symmetries. As we may be able to observe just a single history, it is only thanks to symmetries that we can learn general features of the system from local observations. We have seen, for instance, that if we can observe that “if *h*(5) = *x*, then *h*(6) = *y*”, and the system has the time symmetries of the form ψ_*r*_(*t*) = *t*+*r*, then we can infer the general law that says: “for all *t*, if *h*(*t*) = *x*, then *h*(*t* + 1) = *y*”. Similarly, if we can observe that “conditional on the state being in *Y* at time 5, there is a 50% probability that it will be in *Z* at time 6”, then we can infer the general law that says: “For any *t* in *T*, if the state is in *Y* at time *t*, there is a 50% probability that it will be in *Z* at time *t* + 1.”

However, while the first, non-probabilistic example (where we observe that one state at time 5 is followed by another at time 6) seems unproblematic, the second, probabilistic example is trickier. If we are trapped in a single history, it is unclear how we could ever make an observation such as: “Conditional on the state being in *Y* at time 5, *there is a 50% probability* that it will be in *Z* at time 6.” Making this observation would seem to require looking at many repetitions of states 5 and 6. So, *even if* probabilistic properties *could* be generalized via symmetries once we have observed them, it is unclear how we could observe such properties in the first place.

The solution to this problem lies in the property of *ergodicity*. This is a property that a system may or may not have and that, if present, serves as a prerequisite for inferring probabilistic information from single histories. Indeed, it may be considered a prerequisite for scientific inference more generally. To explain this notion, let us begin with a simple example of how we learn probabilistic information from observing just a single history. Consider a system whose state at any time is the outcome of an independent coin toss, where *T* = {1, 2, 3,…}. So, the state space is *X* = {*Heads*, *Tails*}, and each possible history in Ω is one possible *Heads*/*Tails* sequence.

Suppose the true conditional probability structure on Ω is induced by the single parameter *p*, the probability of *Heads*. In this example, the Law of Large Numbers guarantees that, with probability 1, the limiting frequency of *Heads* in a given history (as time goes to infinity) will match *p*. This means that the subset of Ω consisting of “well-behaved” histories has probability 1, where a history is well-behaved if (i) there exists a limiting frequency of *Heads* for it (i.e., the proportion of *Heads* converges to a well-defined limit as time goes to infinity) and (ii) that limiting frequency is *p*. For this reason, we will almost certainly (with probability 1) get arbitrarily close to the true conditional probability structure on Ω just by observing a single history and counting the number of *Heads* and *Tails* in it.

Now why does this inference work in the present example? As we will see, the system is an example of an *ergodic system*. Its ergodicity manifests itself in the fact that “almost all” histories of the system are “well-behaved”, in the sense that we can read off the desired probability parameter *p* from the limiting frequency of *Heads*.

To define *ergodicity* more precisely, consider again a system with *T* = {1, 2, 3,…} which has all the time symmetries in the set Ψ = {ψ_*r*_: *r* = 0, 1, 2, 3,…} (and perhaps other symmetries as well, though we set these aside for now). Heuristically, the symmetries in Ψ can be interpreted as describing the evolution of the system over time.^27^ Suppose each time-step corresponds to a day. Then the history *h* = (*a*, *b*, *c*, *d*, *e*,*…*) describes a situation where today’s state is *a*, tomorrow’s is *b*, the next day’s is *c*, and so on. Suppose today is Monday. The transformed history ψ_1_(*h*) = (*b*, *c*, *d*, *e*, *f*,…) describes a situation where today’s state is *b*, tomorrow’s is *c*, the following day’s is *d*, and so on. Thus, ψ_1_(*h*) describes the same “world” as *h*, but *as seen from the perspective of Tuesday.* Likewise, ψ_2_(*h*) = (*c*, *d*, *e*, *f*, *g,*…) describes the same “world” as *h*, but *as seen from the perspective of Wednesday*, and so on.^28^

Given the set Ψ of symmetries, an event *E* (a subset of Ω) is Ψ-*invariant* if the inverse image of *E* inside of Ω under ψ is *E* itself, for all ψ in Ψ. Formally, ψ^−1^(*E*) ∩ Ω = *Ε* for all such ψ. Thus, for any history *h* in Ω, *h* is an element of *E* if and only if ψ(*h*) is an element of *E*. For example, suppose again that the elements of *T* represent days, and *E* is the event that some property *P* holds today. If ψ_1_, ψ_2_, ψ_3_, … are the symmetries that shift time by 1 day, by 2 days, by 3 days, and so on, then the Ψ-invariance of *E* implies that property *P* holds today if and only if it holds tomorrow, the day after tomorrow, and so on. Thus, *E* is a “persistent” event: an event one cannot escape from by moving forward in time. In a coin-tossing system, where Ψ is still the set of time translations, examples of Ψ-invariant events are “all *Heads*”, where *E* contains only the history (*Heads*, *Heads*, *Heads*, …), and “all *Tails*”, where *E* contains only the history (*Tails*, *Tails*, *Tails*, …).

Recall that symmetries preserve the unconditional probabilities of any event *E*. The system is *ergodic* (with respect to Ψ) if, for any Ψ-invariant event *E*, the unconditional probability of *E*, i.e., *Pr*_Ω_(*E*), is either 0 or 1.^29^ In other words, the only persistent events are those which occur in *almost no* history (i.e., *Pr*_Ω_(*E*) = 0) and those which occur in *almost every* history (i.e., *Pr*_Ω_(*E*) = 1).^30^ The ergodicity of our coin-tossing system is exemplified by the fact that the Ψ-invariant events “all *Heads*” and “all *Tails*” occur with probability 0.

In an ergodic system, it is possible to estimate the probability of any event “empirically”, by counting the frequency with which that event occurs, much like the probability of *Heads* in the coin-tossing example.^31^ Frequencies are thus evidence for probabilities. The formal statement of this is the following important result from the theory of dynamical systems and stochastic processes.

**Ergodic Theorem:** Suppose the system is ergodic. Let *E* be any event and let *h* be any history. For all times *t* in *T*, let *N*_*t*_ be the number of elements *r* in the set {1, 2,…, *t*} such that ψ_*r*_(*h*) is in *E.* Then, with probability 1, the ratio *N*_*t*_/*t* will converge to *Pr*_Ω_(*E*) as *t* increases towards infinity.^32^

Intuitively, *N*_*t*_ is the number of times the event *E* has “occurred” in history *h* from time 1 up to time *t*. The ratio *N*_*t*_/*t* is therefore the *frequency* of occurrence of event *E* (up to time *t*) in history *h*. This frequency might be measured, for example, by performing a sequence of experiments or observations at times 1, 2,…, *t*. The Ergodic Theorem says that, almost certainly (i.e., with probability 1), the empirical frequency will converge to the *true* probability of *E*, *Pr*_Ω_(*E*), as the number of observations becomes large. The estimation of the probability of *Heads* via the Law of Large Numbers in our coin-tossing example is a special case of this.

To understand the significance of the Ergodic Theorem, let *Y* and *Z* be two subsets of *X*, and suppose *E* is the event “*h*(1) is in *Y*” and *D* is the event “*h*(2) is in *Z*”. Then the intersection *E* ∩ *D* is the event “*h*(1) is in *Y*, and *h*(2) is in *Z*”. The theorem says that, by performing a sequence of observations over time, we can estimate *Pr*_Ω_(*E*) and *Pr*_Ω_(*E* ∩ *D*) with arbitrarily high precision. Thus, we can compute the ratio *Pr*_Ω_(*E* ∩ *D*)/*Pr*_Ω_(*E*) (provided *Pr*_Ω_(*E*) ≠ ∅). But this ratio is the *conditional probability Pr*_*Ε*_(*D*). And so we are able to estimate the conditional probability that the state at time 2 will be in *Z*, given that at time 1 it is in *Y*. This illustrates that, by allowing us to estimate unconditional probabilities, the Ergodic Theorem also allows us to estimate conditional probabilities, and thereby to infer the conditional probability structure {*Pr*_*E*_}_*E*⊆Ω_. Clearly, the system’s symmetries were indispensable for this exercise. Without symmetries, the frequentist reasoning to which the Ergodic Theorem appeals would not make sense.

### Occam’s Razor

We have seen that a system must possess a sufficiently rich set of symmetries to permit general inferences from local observations. Up to now, we have taken for granted that we know, or are justified in hypothesizing, that the system has these symmetries. But what justifies this hypothesis?

This question is crucial for the success of science. Why are we justified in assuming that the system’s laws are the same at different times or in different places? Why should replicability of other scientists’ experimental results be considered the norm, rather than a miraculous exception? Why is it normally safe to assume that the outcomes of experiments will be insensitive to irrelevant details such as the height of the laboratory bench, or the orientation of the apparatus relative to the planet Jupiter?

In effect, we are assuming that the phenomena under investigation are invariant under certain symmetries—both temporal, as discussed earlier, and spatial, as discussed later, including translations, rotations, and so on. But where do we get this assumption from? The answer lies in Occam’s Razor.

Occam’s Razor is generally a principle of parsimony. One of its best-known versions says that, when we try to explain some phenomenon, we should not postulate more entities than strictly explanatorily necessary.^33^ While this version of Occam’s Razor deals with the question of which *entities* to postulate, we are here focusing on another version, which concerns the question of which *regularities* to postulate. Roughly, it says that, if two hypotheses about the regularities in the world are equally consistent with our total evidence, we should prefer the simpler hypothesis.

Now the key point is that the hypothesis of a symmetry-rich system is simpler than the hypothesis of a symmetry-poor system, other things being equal.^34^ To see why this is the case, contrast two cases. If you hypothesize that the universe has a very large set of symmetries, you are thereby postulating a very simple universe. By contrast, if you hypothesize that the universe has very few symmetries, you are postulating a very complex universe. The first universe admits a parsimonious description in light of its symmetry-induced regularity, the second does not. This suggests the following provisional formulation of Occam’s Razor principle:

**Occam’s Razor:** Always assume that a system has the largest possible set of symmetries consistent with all facts about the system that we believe to be nomologically necessary.

We must now make this more precise. We begin by explaining what we mean by “facts about the system that we believe to be nomologically necessary”. We represent this by a collection of those histories among the logically possible ones that we have not ruled out as nomologically impossible. We call this collection of histories our *total nomological evidence* about the system. Formally, it is a subset *E* of ${\mathcal {H}}$. It could capture the “hard” constraints that we take the system to satisfy, such that, to the best of our knowledge, any history outside *E* is not permitted by the laws of the system. Of course, we do not *strictly* know that Ω is a subset of *E*. When we empirically study a system, we do not normally know what Ω is. We can at most be certain that *E* overlaps with Ω. We will suppose, however, that we are ready to make the auxiliary assumption that *E* includes, but may be logically weaker than, Ω.

Given this assumption, we are in a position to test the hypothesis that any given transformation of ${\mathcal {H}}$ is a symmetry of our system. Let ψ be such a transformation, and for any *n*, let ψ^*n*^ be the transformation obtained by applying ψ repeatedly, *n* times in a row. For example, if ψ is a rotation about some axis by angle θ, then ψ^*n*^ is the rotation by the angle *n*θ.^35^ For any such transformation ψ^*n*^, we write ψ^−*n*^(*E*) to denote the inverse image in ${\mathcal {H}}$ of *E* under ψ^*n*^. We say that the transformation ψ is *consistent* with the nomological evidence *E* if the intersection

$$ E \cap\uppsi^{ - 1} (E) \cap\uppsi^{ - 2} (E) \cap\uppsi^{ - 3} (E) \cap \ldots $$

is non-empty. This means that *E* does not falsify the hypothesis that ψ is a symmetry of the system.

For example, suppose we are interested in whether electrostatic forces work the same way at all times. We can test this hypothesis by means of Coulomb’s famous “torsion balance” experiment, which measures the electrostatic attraction or repulsion between two charged objects. Suppose we perform the experiment at time *t*_1_ and obtain evidence *E*_1_, and we perform the same experiment again at time *t*_2_ and obtain evidence *E*_2_. Thus, our evidence is summarized by the event *E* = *E*_1_ ∩ *E*_2_. Let ψ be a time symmetry that shifts *t*_1_ to *t*_2_. Then, focusing for simplicity just on the first two terms of the infinite intersection above, we have

$$ E \cap\uppsi^{ - 1} (E) = E_{1} \cap E_{2} \cap\uppsi^{ - 1} (E_{1} ) \cap\uppsi^{ - 1} (E_{2} ). $$

If the experimental results are the same at times *t*_1_ and *t*_2_, then *E*_1_ = ψ^−1^(*E*_2_), and the expression for *E* ∩ ψ^−1^(*E*) simplifies to *E*_1_ ∩ *E*_2_ ∩ ψ^−1^(*E*_1_). Under reasonable assumptions, this is non-empty, meaning that the evidence has not falsified time invariance of electrostatic forces. But if the experimental results at times *t*_1_ and *t*_2_ were different, then *E*_1_ and ψ^−1^(*E*_2_) would be disjoint, and so the intersection *E* ∩ ψ^−1^(*E*) would be empty, which would mean that the evidence is inconsistent with time invariance. As it happens, many thousands of repetitions of Coulomb’s experiment strongly suggest that the intersection is non-empty, and so ψ is a symmetry.

Now our version of Occam’s Razor says that we should postulate as symmetries of our system a maximal monoid of transformations consistent with our evidence. Formally, a monoid Ψ of transformations (where each ψ in Ψ is a function from ${\mathcal {H}}$ into itself) is *consistent* with our total nomological evidence *E* if the intersection

$$  \bigcap\limits_{{\uppsi  \in \Psi }} {\uppsi ^{{ - 1}} (E)}  $$

is non-empty. This is the generalization of the infinite intersection that appeared in our definition of an individual transformation’s consistency with the evidence. Further, a monoid Ψ that is consistent with *E* is *maximal* if no proper superset of Ψ forms a monoid that is also consistent with *E*.

**Occam’s Razor (formal):** Given our total nomological evidence *E* about a temporally evolving system, always assume that the set of symmetries of the system is a maximal monoid Ψ consistent with *E*.

What is the significance of this principle? Recall that we earlier defined Ψ to be the set of *all* symmetries of our temporally evolving system. In practice, we do not know Ψ. A monoid Ψ that passes the test of Occam’s Razor, however, can be viewed as our best guess as to what the true symmetry monoid is. To disambiguate, let Ψ_true_ denote the true symmetry monoid, and let Ψ_hyp_ denote the hypothesized one.

If Ψ_hyp_ is the hypothesized symmetry monoid, and *E* is our total nomological evidence, the intersection

$$  \bigcap\limits_{{\uppsi  \in \Psi_{\rm{hyp}} }} {\uppsi ^{{ - 1}} (E)}  $$

can be viewed as our best guess as to what the set of nomologically possible histories is. It consists of all those histories among the logically possible ones that are not ruled out by the hypothesized symmetry monoid Ψ_hyp_ and the nomological evidence *E*. We call this intersection our *nomological hypothesis* and label it Ω(Ψ_hyp_, *E*).

To see that this construction makes sense, note that, under certain conditions, our nomological hypothesis Ω(Ψ_hyp_, *E*) will reflect the truth about nomological possibility.

**Remark:** If (i) the hypothesized symmetry monoid Ψ_hyp_ is a subset of the true symmetry monoid Ψ_true_, and (ii) *E* is a superset of Ω, then the true set Ω of nomologically possible histories is a subset of Ω(Ψ_hyp_, *E*).

Condition (i) says that we have not postulated any incorrect symmetries, which is compatible with having overlooked some correct symmetries. Condition (ii) says that we have not mistakenly ruled out any nomologically possible histories, which was our auxiliary assumption about our total nomological evidence. If these conditions hold, our nomological hypothesis will indeed be consistent with the truth and will, at most, be logically weaker than the truth.

It is worth explaining the significance of the auxiliary assumption that we have not mistakenly ruled out any nomologically possible histories (i.e., *E* ⊇ Ω). Consider the simple coin-tossing system from Sect. 2.7, where histories are sequences of *Heads* and *Tails*, and time shifts are symmetries. Now consider the event *E* of getting *Heads* at time 1 and *Tails* at time 2. If we treated *E* as our total nomological evidence, this would exclude time shifts as symmetries: the event of getting Heads at time 1 and Tails at time 2 is not invariant under time shifts. The problem is that *E*, in this case, is not a superset of Ω: it excludes histories that are in fact nomologically possible. The notion of “total nomological evidence” that we require is a “cautious” one. The set *E* should exclude only histories that we are confident in deeming nomologically impossible. This is a subtle issue, and a full treatment is beyond the scope of this paper.

In “Appendix C”, we extend the present analysis by offering criteria for choosing a maximal symmetry monoid Ψ consistent with the evidence *E* in case more than one such monoid can be constructed. We suggest that criteria of inferential modesty and informational parsimony should guide that choice in cases of non-uniqueness.

### The role of time

What is the significance of the linear order of the set *T* of times? Why is time ordered in one way, and not in another? Do the laws of a given system “care” about the ordering of time? To put it another way: what does it mean to say that today comes between yesterday and tomorrow? Intuitively, it means this: the events that happened yesterday cannot “directly influence” the events that will happen tomorrow; their influence must be “mediated” by the events that happen today. We now make this claim precise using a standard notion from probability theory: the *Markov property*.^36^

To explain this property, we first introduce the notion of *conditional independence*. Let {*Pr*_*E*_}_*E*⊆Ω_ be a conditional probability structure, and let *D* and *E* be two events (i.e., subsets of Ω). We say that *D* and *E* are *independent* if *Pr*_*D*_(*E*) = *Pr*_Ω_(*E*) and *Pr*_*E*_(*D*) = *Pr*_Ω_(*D*).^37^ Informally, if we interpret probabilities as encoding “information”, this means that learning whether or not *D* has occurred provides no information about whether or not *E* will occur, and vice versa.

To illustrate, recall the simple coin-tossing system from Sect. 2.7. Let *E* and *D* be the events “the outcome at time 1 is *Heads*” and “the outcome at time 2 is *Tails*”. Then *Pr*_Ω_(*E*) = ½ and *Pr*_Ω_(*D*) = ½, assuming for simplicity that *p* = 0.5. Here, the outcome at time 1 has no effect on the outcome at time 2. So, even if we tossed *Heads* at time 1, this would not change the probability of obtaining *Tails* at time 2, and so *Pr*_*E*_(*D*) = ½. Likewise, the outcome at time 2 tells us nothing about what happened at time 1. If we had not observed the outcome at time 1 but obtained the outcome *Tails* at time 2, we would still assign probability ½ to *Heads* at time 1. So, *Pr*_*D*_(*E*) = ½. Thus, the events *E* and *D* are independent.

Now let *C*, *D*, and *E* be three events. We say that *C* and *E* are *conditionally independent*, *given D*, if *Pr*_*C*∩*D*_(*E*) = *Pr*_*D*_(*E*) and *Pr*_*E*∩*D*_(*C*) = *Pr*_*D*_(*C*). Again, if we interpret probabilities as encoding “information”, this means the following. Suppose you already know that *D* has occurred. Then learning whether or not *C* has occurred provides no further information about whether or not *E* will occur, and vice versa.

To illustrate, return again to the coin-tossing example (where *T* = {1, 2, 3,…}) with *p* = 0.5, but suppose we use the tosses of the fair coin to determine the position of a token on an infinite line. We move the token after each coin toss: if we toss *Heads*, we move the token one space to the right, and if we toss *Tails*, we move it one space to the left. Let us represent the position of the token by an integer (either positive or negative); in other words, *X* = {…,− 3, − 2, − 1, 0, 1, 2, 3,…}. Let *x*_*t*_ denote the position of the token at time *t*. Then the rule becomes the following: “If you toss *Heads* at time *t*, then *x*_*t*+1_ = *x*_*t*_ + 1; if you toss *Tails* at time *t*, then *x*_*t*+1_ = *x*_*t*_ − 1.” For simplicity, suppose the coin always starts at position 0 (i.e., *x*_1_ = 0).^38^

If *D* is an event describing the position of the token at time *t*, and *E* is an event describing its position at time *t* + 1, then these two events are not independent. For example, suppose *E* is the event “*x*_6_ = 3”. Then a simple calculation shows that *Pr*_Ω_(*E*) = 5/16. If *D* is the event “*x*_5_ = 2”, then *Pr*_*D*_(*E*) = ½, because the token now has a 50% probability of moving from position 2 to position 3 in one time step. Thus, *Pr*_*D*_(*E*) ≠ *Pr*_Ω_(*E*). The location of the token at time 5 tells us a great deal about its probable location at time 6.

However, once we know the position at time 5, learning the position at time 4 tells us *nothing further* about the position at time 6. Continuing the previous example, let *C* be the event “*x*_4_ = 1”. Then straightforward calculations show that *Pr*_*C*∩*D*_(*E*) = ½ = *Pr*_*D*_(*E*) and *Pr*_*E*∩*D*_(*C*) = ½ = *Pr*_*D*_(*C*). In other words, if we *already knew* that the token’s position was 2 at time 5 (so that it had a 50% probability of moving to position 3 at time 6), then learning its position at time 4 tells us *nothing further* about where it might be at time 6. Likewise, if we *already knew* that the token’s position was 2 at time 5 (so that it has a 50% probability of having been at position 1 at time 4), then learning its position at time 6 tells us *nothing further* about where it might have been at time 4.

In this example, the conditional independence of the events *C* and *E*, given *D*, is due to the fact that *D* concerns the state of the system at a point in time *between* the times described by *C* and *E* and that *D* provides us with *complete* information about the state of the system at this intermediate time. If *D* provided only partial information about that state, we would not get the same result. For example, suppose *D′* is the event, “*x*_5_ = 0, 2, or 4”, which does not fully specify the state at time 5. Then it can be shown that *Pr*_*C*∩*D′*_ (*E*) > *Pr*_*D′*_ (*E*). Here, learning additional information about the state at time 4 can still tell us something about where the coin is likely to be at time 6.

Now let us generalize this example. Let *T* be any linearly ordered set, let *X* be any set of states, and consider a temporally evolving system given by a collection Ω of possible histories (i.e., functions from *T* into *X*) and a conditional probability structure {*Pr*_*E*_}_*E*⊆Ω_. For any time *t* in *T*, and any state *x* in *X*, let *E*_*x*_^*t*^ denote the event “the state of the system at time *t* is *x*”. More generally, for any subset *Y* of *X*, let *E*_*Y*_^*t*^ denote the event “the state of the system at time *t* is an element of *Y*”. We say that the system satisfies the *Markov property* if, for any times *r* < *s* < *t* in *T*, any subsets *Y* and *Z* of *X*, and any state *x* in *X*, the events *E*_*Y*_^*r*^ and *E*_*Z*_^*t*^ are conditionally independent, given the event *E*_*x*_^*s*^. In other words, if you have *complete information* about the state of the system at some time *s* (you know that the state is *x*), then learning something about its state at some earlier time (e.g., that it was an element of *Y* at time *r*) tells you *nothing further* about its probable state at some later time (e.g., about how probable it is that it will fall into the set *Z* at time *t*). Roughly speaking, this means that the state of the system at time *r* cannot “directly influence” the state of the system at time *t*. It can only influence that state “indirectly”, via influencing the state at the intermediate time *s*. Any system with this property is called *Markovian*.

Note that the Markov property does not say that the system’s future evolution is unconditionally independent of its past. It just says that the dependency of the future on the past is mediated through the present. This property is fundamental to the way we normally think about time. To see this, imagine a universe where the Markov property was *not* true. Then there would exist some times *r* < *s* < *t* in *T*, some subsets *Y* and *Z* of *X*, and some state *x* in *X*, such that the conditional probability *Pr*(*E*_*Z*_^*t*^ | *E*_*Y*_^*r*^∩*E*_*x*_^*s*^) is distinct from *Pr*(*E*_*Z*_^*t*^ | *E*_*x*_^*s*^).^39^ In other words, even with a *complete* specification of the present state *x*, the probability of some *future* event *Z* would depend on whether or not some *past* event *Y* had occurred. This would suggest that the state specification *x* does *not*, in fact, contain all the information about the system’s present state; somehow, information about the past is bypassing the present and “leaking” directly into the future. This, in turn, suggests that this so-called “past” is not really in the past at all; our model of the system’s time structure is incorrect.

We take the Markov property to be a necessary condition for the “correct” ordering of time. To be “well-behaved”, a temporally evolving system must be Markovian. What the present must do at any point in time in order to *count* as the present is “separate” the past from the future. If this property is violated, the set *T* does not properly play the role of time.

Three points are worth noting. First, some systems may admit multiple time orderings with respect to which they are Markovian. An extreme limiting case is given by our original coin-tossing system without the moving token, which is Markovian with respect to *every* ordering of *T*. Here, the precise order of time is irrelevant. By contrast, in the modified coin-tossing system with the token, the order of time matters, as we have seen. In fact, the temporal order with respect to which the system satisfies the Markov property is essentially unique; it is unique up to time reversals. This brings us to our second point. Although the Markov property says something about the linear “topology” of time, it tells us nothing about the *direction* of time. As illustrated by the modified coin-tossing system, the Markov property is completely invariant under time reversals. In other words, the Markov property only says that the present separates the past from the future. But it does not tell us on which side of the present lies the past, and on which side lies the future. And third, just as the Markov property says nothing about the direction of time, so it says nothing about its duration. There is no purely Markovian way of measuring the “length” of a time interval or saying when one time interval is longer than another.

What, then, can we say about the directionality and length of time? It turns out that symmetries are crucial for the analysis of both. In the case of length, we offer a detailed analysis in Sect. 3.9, showing that there is a natural way of measuring time duration, as long as the system has sufficiently rich symmetries. And in the case of directionality, we can say that a condition for time to have a direction is that time reversals are *not* symmetries of the system. Since time reversals are symmetries of classical mechanical systems (in the sense explained in footnote 19), it follows that, in those systems, there is no real direction of time: temporal orders are unique at most up to time reversal. By contrast, in thermodynamic systems, time reversals are not symmetries, and hence these systems meet the condition for time to have a direction. To the extent that the world, as seen from our perspective, is best understood as a system in which time reversals are not symmetries, there is then a coherent basis for the directionality of time (for further discussion, see Roberts 2013).

## Spatially extended systems

### Basic definitions

We now turn to a more richly described class of systems whose states evolve over time. To define a system in this class, we still represent *time* by a linearly ordered set *T*, but also incorporate an explicit notion of *space*, represented by a set *S* of *spatial locations*. Let *S* × *T* be the set of all ordered pairs of the form (*s*, *t*), where *s* is an element of *S*, and *t* is an element of *T*. We refer to *S* × *T* as *space*–*time*. Again, let *X* denote a set of possible states, called the *state space*. Unlike before, the elements of *X* are no longer “global” states, in which the system can be *at specific points in time*, but “local” states, in which the system can be *at specific points in space and time*. Again, we treat the elements of *X* as primitives of our model. Histories are now functions from space–time (rather than merely time) into the state space. Formally, a *spatially extended history* is a function *h* from *S* × *T* into *X*. For each point (*s*, *t*) in *S* × *T*, *h*(*s*, *t*) is the state of the system in spatial location *s* at time *t*.

In analogy to our earlier model, we write Ω to denote the set of all spatially extended histories deemed *possible*, which, as before, play the role of possible worlds. Again, this is a subset—often a proper one—of the set $\mathcal{{H}}$ of all *logically possible* histories (here, all functions from *S* × *T* into *X*). So, membership in Ω is best interpreted as *nomological possibility*. Subsets of Ω are called *events*.

Finally, we define a *conditional probability structure* on Ω. As before, this is a family of conditional probability functions {*Pr*_*E*_}_*E*⊆Ω_, containing one *Pr*_*E*_ for each event *E* in Ω, with standard properties. Recall that *Pr*_*E*_ assigns to any event in Ω the conditional probability of that event, given *E*. A *spatially extended system* is the pair consisting of the set Ω of possible spatially extended histories and the conditional probability structure {*Pr*_*E*_}_*E*⊆Ω_.

For example, in a classical mechanical system, *T* is the set **R** of real numbers, *S* is the three-dimensional Euclidean space (i.e., *S* = **R**^3^), and each state *h*(*s*, *t*) in *X* is given by the set of particles present at spatial location *s* at time *t*, along with their physically relevant properties (e.g., masses and momenta) and the values of any force fields (e.g., gravity) acting on these particles.^40^ In a classical electrodynamical system, the state *h*(*s*, *t*) must also specify the particles’ charges, along with the electric and magnetic field vectors at (*s*, *t*). In that sense, electrodynamics relies on a richer ontology than classical mechanics.

In a quantum–mechanical system, it might be tempting to suppose that *S* = **R**^3^, and to suppose that *h*(*s*, *t*) is given by the values of the wave functions of each of the particles in the system at space–time location (*s*, *t*). But this is not correct, because the wave functions of interacting particles in a quantum system cannot generally be defined independently of each other. Instead, we must define a *joint* wave function for the entire multi-particle system. So, in a quantum–mechanical system with *n* particles, we would define space to be *S* = (**R**^3^)^*n*^, with three coordinates representing the spatial “position” of each of the *n* particles in an underlying ordinary Euclidean space^41^; and we would define the set *X* of possible states of the system to be the set of complex numbers, capturing amplitudes, whose squared absolute values behave formally like probabilities. Thus a spatially extended history *h* is a function from (**R**^3^)^*n*^ × *T* into the set of complex numbers, representing the joint wave function of the whole ensemble of particles.

For instance, if there are two particles, labelled 1 and 2, then *h*(*x*_1_, *y*_1_, *z*_1_, *x*_2_, *y*_2_, *z*_2_, *t*) represents the joint state at time *t* of particles 1 and 2 at positions *x*_1_, *y*_1_, *z*_1_ and *x*_2_, *y*_2_, *z*_2_ in the underlying three-dimensional Euclidean space. This joint state of the two particles is a complex number whose squared absolute value can be interpreted, under some assumptions, as the probability of particles 1 and 2 being observable at positions *x*_1_, *y*_1_, *z*_1_ and *x*_2_, *y*_2_, *z*_2_, respectively, at time *t*.

### Determinism and indeterminism

As in the case of temporally evolving systems, we can define a family of notions of determinism and indeterminism for spatially extended systems. For any subset *L* of locations in *S* × *T*, we write *h*_*L*_ to denote the restriction of the function *h* to the points in *L*. We can then ask for which proper subsets *L* of *S* × *T*, if any, *h*_*L*_ has a unique extension to all of *S* × *T* in Ω. Again, an *extension* of *h*_*L*_ is a history *h′* such that $h^{{\prime }} _{L} = h_{L}$. When *h*_*L*_ is uniquely extendible to all of *S* × *T*, we say that history *h* is *L*-*deterministic*.

For example, the histories of classical mechanical systems are *L*-deterministic for any subset *L* of *S* × *T* that has the form *S* × *T′* where *T′* is any non-empty subset of *T*. Information about the system for even a single “time slice” of space–time, i.e., a set of the form *S* × {*t*} for some *t* in *T*, suffices to determine the full spatially extended history. In contrast, the histories of quantum–mechanical systems (if wave-function collapses are allowed) are not generally *L*-deterministic when *L* consists of time slices.

The present definitions allow us to explore some interesting possibilities not captured by standard definitions that focus exclusively on past-to-future determination.^42^ For example, some systems might encode their entire spatially extended history in each individual space–time location. Histories would then be *L*-deterministic for every singleton set *L* = {(*s*, *t*)}, where (*s*, *t*) is in *S* ×  *T*. Here, we would have an extreme form of local-to-global determinism. Alternatively, some systems might encode their entire spatially extended history in some collection of “spatial slices of time”, i.e., some subset *L* of *S* × *T* which has the form *S′* × *T*, where *S′* is a non-empty subset of *S*, possibly singleton. This would be a kind of spatial, not temporal, determinism.^43^ Other systems might never be *L*-deterministic for any proper subset *L* of *S* × *T*.

There may also be some more limited, non-global forms of determination, for instance when a history restricted to some set *L* of locations is uniquely extendible to a history restricted to some superset L* of *L*, which is still smaller than *S* × *T* in its entirety.^44^ To capture this idea, we can say that a history *h* is *L*-to-L^*^-*deterministic* if, for any history *h′* in Ω, if $h_{L} = h^{{\prime }} _{L}$, then $h_{L^{*}} = h^{{\prime }} _{L^{*}}$.

We might imagine, for instance, systems that are deterministic “across space” but not “across time”. In such a system, a history restricted to some set *L* of the form *S′* × {*t*}, where *S′* is a non-empty subset of *S* and *t* a point in time, might determine the entire “time slice” of that history across L^*^ = *S* × {*t*}, but not the rest of the history. Some crystals and other chemical or physical systems involving highly regular spatial structures might have this feature. Similarly, for suitable specifications of *L* and L^*^, we can represent the phenomenon that, in some systems in which “information” travels with finite speed, events at particular space–time locations at time *t*_1_ are entirely determined by the events occurring within their “backwards light cones” at some time *t*_0_ < *t*_1_. Such systems may be *L*-to-L^*^-deterministic, but not deterministic in a more global sense.

### Nomological possibility and necessity

In analogy to the case of temporally evolving systems, we can define two modal operators for each set *L* of space–time locations, namely nomological possibility and necessity relative to *L*. For each set *L* ⊆ *S* × *T*, call one history, *h′*, *accessible* from another, *h*, relative to *L*, if the restrictions of *h* and *h′* to *L* coincide, i.e., $h^{{\prime }} _{L} = h_{L}$. We then write *hR*_*L*_*h′*. For any event *E* ⊆ Ω, we define

- ◆_*L*_ *E* = {*h* ∈ Ω: for some *h′* ∈ Ω with *hR*_*L*_*h′*, we have *h′* ∈ *E*},
- ■_*L*_ *E* = {*h* ∈ Ω: for all *h′* ∈ Ω with *hR*_*L*_*h′*, we have *h′* ∈ *E*}.

Here, ◆_*L*_ *E* and ■_*L*_ *E* are, respectively, the sets of all histories in which *E* is nomologically possible and nomologically necessary *once the history in space*–*time region L is given*. Important special cases are (i) $L =   S \times T^{\prime}$, where *S* is all of space and $T^{\prime}$ is a particular set of time points, such as those up to time *t*, (ii) *L* = *S′* × *T*, where *T* is all of time and *S′* is some spatial region, and (iii) *L* = ∅ for possibility and necessity in the “atemporal” sense. Since the present definitions are completely analogous to their earlier counterparts in Sect. 2.3, we will not say more about them here.

### Modal and probabilistic properties

We now turn again to the question of how to distinguish between those properties of a system that qualify as “laws” and those that fall short of being laws. As before, our analysis is based on the notion of *symmetry*, but now with the additional ingredient that these symmetries can involve space as well as time.

In analogy to our earlier definition, a *property of histories*, *P*, is a binary feature that a spatially extended history may or may not have. Its *extension* is some subset [*P*] of the set $\mathcal{{H}}$ of all logically possible histories. A spatially extended history *h* satisfies *P* if *h* belongs to [*P*]. Again, if [*P*] includes all of Ω, then *P* can be called *nomologically necessary.* Similarly, a *probabilistic property*, $\mathcal{{P}}$, is a binary feature that a conditional probability structure may or may not have, and its extension, [$\mathcal{{P}}$], is the set of all those conditional probability structures on Ω that satisfy $\mathcal{{P}}$.

### Symmetries

The notion of a *state symmetry* for spatially extended systems is virtually identical to the one defined in Sect. 2.5 for temporally evolving systems, so we do not discuss it further.^45^ Instead, we turn directly to *symmetries acting on space*–*time*. Let ψ be a function from *S* × *T* into itself (i.e., a transformation of space–time). Again, ψ induces a function from the set $\mathcal{{H}}$ of logically possible histories into itself. For any spatially extended history *h*, we define the transformed history

$$ \uppsi(h) = h^{{\prime }} ,\;{\text{where, for all}}\;(s,t)\;{\text{in}}\;S \times T,h^{{\prime }} (s,t) = h(\uppsi(s,t)). $$

As before, given any set *E* of histories in $\mathcal{{H}}$, the *inverse image* of *E* under ψ, written ψ^−1^(*E*), is the set of all histories *h* in $\mathcal{{H}}$ such that ψ(*h*) lies in *E*. The function ψ is a *symmetry* if

- ψ(Ω) = Ω; and
- for any events *E* and *D* in Ω, if *E′* and *D′* are the inverse images of *E* and *D* under ψ, then *Pr*_*E′*_ (*D′*) = *Pr*_*E*_(*D*).^46^

For example, if *T* is the set of real numbers (i.e., *T* = **R**) and *S* is the three-dimensional Euclidean space (i.e., *S* = **R**^3^), we can consider a spatially extended system in classical mechanics. The following transformations of *S* × *T* are space–time symmetries of such a system, each defined for all (*s*, *t*) in *S* × *T*:

- *Time translation*: ψ(*s*, *t*) = (*s*, *t* + *r*), where *r* is a fixed real number;
- *Spatial translation*: ψ(*s*, *t*) = (*s* + *v*, *t*), where *v* is a fixed three-dimensional vector (an element of **R**^3^); and
- *Space*–*time rescaling*: ψ(*s*, *t*) = (*r s, r t*), where *r* > 0 is a fixed real number.

More general symmetries include composite functions resulting from the combination of a transformation ϕ of the state space (*X*) with a transformation ψ of space–time (*S* × *T*).^47^ Examples in classical mechanics are spatial rotations, spatial reflections, spatial rescalings, and Galilean transformations.^48^ Crucially, it is possible that neither the transformation ϕ of the state space nor the transformation ψ of space–time alone is a symmetry, and yet, when combined, they form a symmetry.^49^

Of course, any combination of symmetries is also a symmetry. An example is a *spatiotemporal translation*, which is a combination of a time translation and a spatial translation. In a classical electrodynamical system, *only* the spatiotemporal translations and rotations are space–time symmetries. Galilean transformations are not space–time symmetries of classical electrodynamics; indeed, this was the original impetus for the development of special relativity theory.

### Laws and their significance

As in the earlier case of temporally evolving systems, a *modal law* of a spatially extended system is a property of histories, *P*, that is nomologically necessary for the system and invariant under all of the system’s symmetries. A *probabilistic law* is a probabilistic property, $\mathcal{{P}}$, that is satisfied by the system’s conditional probability structure and invariant under all of its symmetries.

For example, let *S* = **R**^3^ and *T* = **R**, and suppose the symmetry monoid Ψ contains all the spatiotemporal translations defined in the previous section. Suppose all histories of the system satisfy the property *P* which says: “If the state at space–time position (3, 7, 2, 14) is *x*, then at position (4, 8, 1, 17) it is *y*.” If ψ is a spatial translation by the vector (1, 2, 3), then the inverse image of [*P*] under ψ corresponds to the property *P′* which says: “If the state at (4, 9, 5, 14) is *x*, then at position (5, 10, 4, 17) it is *y*.” Clearly, [*P′*] is not the same as [*P*], and so property *P* falls short of being a law.

However, suppose all histories satisfy the property *P* which says: “For any location (*s*_1_, *s*_2_, *s*_3_) in *S* and any time *t* in *T*, if the state at space–time position (*s*_1_, *s*_2_, *s*_3_, *t*) is *x*, then at position (*s*_1_ + 1, *s*_2_ + 1, *s*_3_ − 1, *t* + 3) it is *y*.” It is easy to see that [*P*] *is* invariant under all spatiotemporal translations. If Ψ consists *only* of the spatiotemporal translations, then *P* is invariant under *all* symmetries, and so *P* is a law.

An illustration is *Gauss’s Law* in an electrodynamical system. This asserts, roughly, that the net “flux” of the electric field passing through the walls of any closed compartment is proportional to the net charge contained inside that compartment. This property is invariant under spatiotemporal translations, because the net flux and the net charge are unchanged by such transformations. Indeed, Gauss’s Law is preserved by *every* symmetry of an electrodynamical system; that is why it is a law.

As before, the significance of laws, as opposed to properties that fall short of being laws, lies in their openness to testing and generalization. Consider again the property: “If the state at space–time position (3, 7, 2, 14) is *x*, then at position (4, 8, 1, 17) it is *y*.” This property is observable exactly once in any history, namely at space–time position (3, 7, 2, 14) alone. Taken in isolation, the observation that some history has this property tells us very little. It leaves open whether there is some broader regularity. By contrast, consider the property: “For any location $(s_{1} ,s_{2} ,s_{3} )$ in *S* and any time *t* in *T*, if the state at position $(s_{1} ,s_{2} ,s_{3} ,t)$ is *x*, then at position $(s_{1} + 1,s_{2} + 1,s_{3} - 1,t + 3)$ it is *y*.” Recall that, if the system’s symmetry monoid consists of all spatiotemporal translations, then this property is a law. Indeed, it has many observable manifestations in each history, both at different times and in different places, and it thus picks up a pattern that we can in principle test and use as a basis for predictions, even within a single history.

### Spatiotemporal ergodicity and its significance

Before turning to a more detailed analysis of the role of space in a spatially extended system, it is worth sketching how the property of ergodicity can be extended to such a system and discussing the significance of this. In the present case, too, ergodicity is the key to learning a system’s conditional probability structure, even if we are able to observe only a single history of the system.

Recall that, for some set Ψ of symmetries, an event *E* (a subset of Ω) is Ψ-*invariant* if, for every ψ in Ψ, the inverse image of *E* inside of Ω under ψ is *E* itself. For illustrative purposes, suppose Ψ consists of all spatiotemporal translations by four-dimensional vectors of integers (applying the definition from Sect. 3.5).^50^ The system is *spatiotemporally ergodic* if the unconditional probability of any Ψ-invariant event *E*, *Pr*_Ω_(*E*), is either 0 or 1.

Since Ψ consists of spatiotemporal translations, Ψ-invariant events are events from which one cannot escape by travelling through space, or by travelling forwards or backwards through time. In our example, let ψ be a spatiotemporal translation in Ψ such that, for all (*s*_1_, *s*_2_, *s*_3_, *t*) in *S* × *T*, we have $\uppsi(s_{1} ,s_{2} ,s_{3} ,t) = (s_{1} + 5,s_{2} - 7,s_{3} + 10,t + 3)$. If we interpret the spatially extended history *h* as describing a possible world “from the perspective of position (0, 0, 0, 0)”, then, heuristically, the transformed history ψ(*h*) describes the same world “from the perspective of position (5, − 7, 10, 3)”. Here a Ψ-invariant event *E* has the property that whenever a history *h* is in *E*, then so is ψ(*h*). Roughly speaking, this means that the world described by *h* appears to be in the set *E* “from the perspective of position (0, 0, 0, 0)” if and only if it appears to be in *E* “from the perspective of position (5, − 7, 10, 3)”, and so on. Ergodicity requires any such event to occur either almost always (with probability 1) or almost never (with probability 0).

In a spatiotemporally ergodic system, we can estimate the probability of any event by counting the spatiotemporal frequency with which that event occurs.

**Spatiotemporal Ergodic Theorem:** Suppose the system is spatiotemporally ergodic. Let *E* be any event and let *h* be any history. For all *r* > 0, let Ψ_*r*_ be the set of all spatiotemporal translations by any vector (*v*_1_, *v*_2_, *v*_3_, *v*_4_) with integer coordinates between 1 and *r*. Let *N*_*r*_ be the number of translations ψ in Ψ_*r*_ such that ψ(*h*) is in *E.* Then, with probability 1, the ratio *N*_*r*_/*r*^4^ will converge to *Pr*_Ω_(*E*) as *r* increases towards infinity.^51^

Intuitively, *N*_*r*_ is the number of times the event *E* has “occurred” in the spatially extended history *h* from time 1 to time *r* and inside a three-dimensional box with side-length *r.* The ratio *N*_*r*_/*r*^4^ is therefore the *frequency* of occurrence of event *E*, up to time *r* inside this box, in the spatially extended history *h*. This frequency might be measured, for example, by performing a sequence of experiments or observations inside this box. The Spatiotemporal Ergodic Theorem says that, with probability 1, the empirical frequency will converge to the true probability of *E* as the number of observations becomes large.^52^ As explained in Sect. 2.7, we can use this procedure to estimate not only unconditional probabilities but also conditional ones, and thereby to learn the properties of the conditional probability structure {*Pr*_*E*_}_*E*⊆Ω_.

A broader lesson is that whether a system is ergodic in the first place depends on the system’s symmetries. If a system is rich in symmetries, then ergodicity becomes easier to achieve than if the system has only few symmetries. To see this, note that the notion of Ψ-invariance is logically more demanding for a larger set Ψ of symmetries than for a smaller one, since an event *E* will need to be preserved under more symmetries in order to qualify as Ψ-invariant. As a result, there will be fewer Ψ-invariant events if Ψ is large, and hence the property of ergodicity, which constrains the probability of Ψ-invariant events, becomes less demanding. Conversely, if the set Ψ of symmetries is small, more events may qualify as Ψ-invariant. In the limit, if Ψ contains only the (trivial) identity symmetry, then *every* event *E* will be Ψ-invariant, and so *no* system with a non-degenerate conditional probability structure will qualify as ergodic. (Recall that, in an ergodic system, the unconditional probability of every Ψ-invariant event must be either 0 or 1. If all events are Ψ-invariant, this rules out non-degenerate probabilities.) Thus, we must conclude not only that ergodicity is a key prerequisite for inferring a system’s conditional probability structure from local observations, but also that without enough symmetries this inference would not get off the ground.^53^

### The role of space

What is the role of “space” in a spatially extended system? As we will now see, the structure of space affects the way the system evolves over time. To make this precise, we first introduce a formal representation of the topology of space and then discuss its role in the system’s dynamics.

The topology of space can be represented by a binary relation → between subsets of *S*. Heuristically, if *R* and *R′* are two subsets of *S*, such as two “regions” of space, then *R* → *R′* means that *R* and *R′* are “adjacent” in that information from *R* can flow “directly” into *R′*, without needing to pass through some intervening points “between” *R* and *R′*. Later, we explain exactly what we mean by “information flow”, but for our initial discussion, we leave it unexamined. We call → the *adjacency structure* of space.^54^

Adjacency structures arise naturally in many systems. For example, suppose *S* is ordinary three-dimensional Euclidean space, and suppose information can flow only “continuously” through this space. This would be the case, for instance, in a system consisting of particles travelling along continuous trajectories and interacting via continuous force fields, such as those found in classical mechanics, or in a system described by partial differential equations, such as those found in quantum mechanics, classical electrodynamics, or hydrodynamics. In such systems, for any subsets *R* and *R′* of *S*, we have *R* → *R′* if there exists a point *s* in *R* such that, for any radius *r* > 0, the ball of radius *r* centred at *s* intersects *R′*.^55^

For another example, suppose *S* is the *three*-*dimensional integer lattice*: the set of all ordered triples $s = (s_{1} ,s_{2} ,s_{3} )$, where *s*_1_, *s*_2_, and *s*_3_ are integers. Say that two points *s* and *s′* in *S* are *neighbours* if they differ in only one coordinate and that difference is 1. Thus (3, 7, 5) and (3, 6, 5) are neighbours. Suppose information can flow only directly between neighbours in the lattice. Then, for any subsets *R* and *R′* of *S*, we have *R* → *R′* if some point in *R* is a neighbour of some point in *R′*.^56^ Discrete spatial geometries of this kind can be found in a class of systems called *cellular automata*.^57^

For a final example, consider a *directed graph*, which consists of a set of “vertices”, along with a set of “arrows” which connect pairs of vertices. Directed graphs can be used to model electric circuits, communication networks (e.g., the internet), economic and transportation networks, and biological systems (e.g., neural networks, gene regulatory networks, and epidemiological networks). Suppose *S* is the set of vertices. Then, for any subsets *R* and *R′* in *S*, we have *R* → *R′* if there is an arrow from some vertex in *R* to some vertex in *R′*.

If the sets *R* and *R′* overlap (i.e., *R* ∩ *R′* ≠ ∅), then clearly we have both *R* → *R′* and *R′* → *R*. However, the examples above show that we can have *R* → *R′* even if *R* and *R′* do not overlap, as long as the two sets “touch” each other in some sense. Intuitively, *R* → *R′* means that it is not possible to interpose any “barrier” between *R* and *R′*; there is no “gap” between them.

What role does the adjacency structure play in a spatially extended system? Why does space have one adjacency structure rather than another? Just as we argued earlier in the case of time, we will now argue that a “correct” adjacency structure on space is one that satisfies a Markov property with respect to the conditional probability structure {*Pr*_*E*_}_*E*⊆Ω_. This Markov property is defined by considering conditional probabilities based on “partial information” about a spatially extended history.

We therefore need a precise way to talk about such “partial information”. Let *R* be a subset of *S*, and let *R* × *T* be the set of all ordered pairs (*s*, *t*), where *s* is an element of *R*, and *t* is an element of *T*. So, *R* × *T* is the set of all time-slices restricted to the spatial region *R*. For any history *h* in Ω, recall that $h_{R \times T}$ denotes the restriction of *h* to the set *R* × *T.* This restriction records only the part of the history *h* which “happens inside *R*”. Let us then define the event $[ h_{R \times T}]$ to be the set of all extensions of $h_{R \times T}$ to full histories in Ω, i.e., the set of *all h′* in Ω such that $h_{R \times T} = h_{R \times T}^{{\prime }}$. These are precisely the histories that are accessible from *h* relative to the space–time region *R* × *T*. The Markov property for adjacency structures will be based on conditional independence with respect to such events, in the following way.

For any event *E* (i.e., a subset of Ω), we say that *E happens inside R* if, for all histories *h* and *h′* such that $h_{R \times T} = h_{R \times T}^{{\prime }}$, history *h* is an element of *E* if and only if history *h′* is an element of *E.* In other words, the question of whether or not a particular history is an element of *E* is completely determined by the restriction of that history to spatial “region” *R*.

A *tripartition* of *S* is a triple (*R*, *R′*, *R″*), where *R*, *R′*, and *R″* are three disjoint subsets of *S* which together cover *S* (i.e., *R* ∪ *R′* ∪ *R″* = *S*), such that it is *not* the case that *R* → *R″* or *R″* → *R*. Heuristically, this means that the set *R′* “separates” *R* from *R″*. For example, suppose *S* is three-dimensional Euclidean space, with the adjacency structure described above. Let *R* be the set of all points whose distance to the origin is less than 1: the *unit ball*. Let *R′* be the set of all points whose distance to the origin is between 1 and 2, so *R′* is a sort of thick spherical “shell” around *R*. Finally, let *R″* be the set of all points whose distance to the origin is greater than 2. Then (*R*, *R′*, *R″*) is a tripartition of *S*.

We say that the adjacency structure → satisfies the *Markov property* with respect to the conditional probability structure {*Pr*_*E*_}_*E*⊆Ω_ if, for any tripartition (*R*, *R′*, *R″*) and any history *h* in Ω, any event which happens inside *R* is conditionally independent from any event which happens inside *R″*, given everything that happens in *R′* (i.e., given $[h_{R^{\prime} \times T}]$). Heuristically, this means that there is no way for information to propagate from *R* into *R″*, or vice versa, without first passing through *R′*. For example, suppose *S* is three-dimensional Euclidean space, and (*R*, *R′*, *R″*) is the “concentric sphere” tripartition described above. In this case, the spherical shell *R′* acts as a barrier that isolates the ball-shaped compartment *R* from any influences coming from the “outer region” *R″*. If we have complete information about the history inside *R′* (i.e., we know $[h_{R^{\prime} \times T}]$), then we have complete control over the boundary conditions for any experiment we conduct inside *R*, and thus we do not need to control or even know what happens in the outer region *R″*.

Scientists implicitly assume that space satisfies the Markov property every time they construct a laboratory apparatus that “isolates” some experiment from the surrounding environment. Indeed, people also implicitly assume the Markov property every time they close the doors and windows of their houses to keep out the cold. Thus, the Markov property is central to the way we ordinarily think of space. It underpins the adjacency structure of space in the same way it underpins the order structure of time.

Just as with time, however, the Markov property does not *completely* determine the structure of space. First, there may be more than one adjacency structure on *S* which satisfies the Markov property with respect to {*Pr*_*E*_}_*E*⊆Ω_, just as there may be more than one Markovian order on *T*. Second, the adjacency structure alone leaves many important geometric properties of *S* unspecified. For example, in many contexts, we would like to define a *metric* on *S*, which determines a notion of “distance” between points. This is obviously crucial in classical mechanics, for example. The adjacency structure does not determine a unique metric. We therefore now turn to the question of how we might arrive at such a metric.

### Duration and distance

Recall that the set *T* of times is linearly ordered. In many contexts, we would like to define a notion of *duration* on *T*. That is, given four moments *t*_1_, *t*_2_, *t*_3_, and *t*_4_ in *T*, with *t*_1_ < *t*_2_ and *t*_3_ < *t*_4_, we would like to determine whether the time interval between *t*_1_ and *t*_2_ is greater or smaller than that between *t*_3_ and *t*_4_. To do this, we suppose that the monoid of temporal symmetries, Ψ, acts *freely and transitively* on *T*, and all symmetries in Ψ are *order*-*preserving*. This means that, for any times *t*_1_ and *t*_2_ in *T*, there is a *unique* symmetry ψ in Ψ such that ψ(*t*_1_) = *t*_2_, and, for any symmetry ψ in Ψ, *t*_1_ < *t*_2_ implies ψ(*t*_1_) < ψ(*t*_2_). We can then define a formal “subtraction” operation on *T* as follows. Fix some reference time *t*_0_. Now, for any times *t*_1_ and *t*_2_ in *T*, we define

$$ t_{2} - t_{1} =\uppsi(t_{2} ),{\text{where}}\;\uppsi\;{\text{is}}\;{\text{the}}\;{\text{unique}}\;{\text{temporal}}\;{\text{symmetry}}\;{\text{in}}\;\Psi \;{\text{such}}\;{\text{that}}\;\uppsi(t_{1} ) = t_{0} . $$

In particular, this implies that *t* − *t*_0_ = *t*, for any *t* in *T*. For any four points *t*_1_, *t*_2_, *t*_3_, and *t*_4_ in *T*, we say that the time interval from *t*_1_ to *t*_2_ is greater than the one from *t*_3_ to *t*_4_ if *t*_2_ − *t*_1_ > *t*_4_ − *t*_3_. Similarly, we can define a formal “addition” operation on *T*. For any times *t*_1_ and *t*_2_ in *T*, we define

$$ t_{1} + t_{2} =\uppsi(t_{2} ),{\text{where}}\;\uppsi\;{\text{is}}\;{\text{the}}\;{\text{unique}}\;{\text{temporal}}\;{\text{symmetry}}\;{\text{in}}\;\Psi \;{\text{such}}\;{\text{that}}\;\uppsi(t_{0} ) = t_{1} . $$

The set *T*, with the ordering < and the operation +, forms a *left*-*linearly ordered group*.^58^

In many contexts, we would also like to define a *metric* on *S*, which determines a notion of “distance” between points in space. As we have noted, the adjacency structure does not determine a unique metric. But we can define a concept of distance on *S* by measuring how long it takes for information to travel from one point to the other. To do this, we need to use the concept of duration we have just introduced.

Given any two regions *R* and *R′* of *S*, and a time *t* in *T*, we define what it means for region *R′* to be “not reachable” from region *R* in time *t*. We begin with some preliminary definitions. For any subset *R* of *S*, and any time *t* in *T*, let *R* × {*t*} denote the set {(*s*, *t*): *s*∈*R*}. Adapting our earlier definition, we say that an event *E happens inside R* *at* *time* *t* if, for all histories *h* and *h′* such that $h_{{R \times \{ t\} }}  = h_{{R \times \{ t\} }}^{{\prime }}$, history *h* is an element of *E* if and only if history *h′* is an element of *E.* In other words, whether or not a particular history is an element of *E* is completely determined by the restriction of that history to the space–time region *R* × {*t*}. Further, let *R*^C^ denote the *complement* of *R* in *S*, i.e., *R*^C^ = {*s* ∈ *S*: *s* ∉ *R*}. Given any two subsets *R* and *R′* of *S*, and a time *t* in *T* with *t* > *t*_0_, we now say that *R′* is *not reachable from R in time t* if, for any history *h* in Ω, any event which happens in *R′* at time *t* is conditionally independent of any event which happens in *R* at *t*_0_, given $[h_{{R^{C}  \times \{ t_{0}\} }} ]$. Informally, once we have complete information about the state of the system *outside* the set *R* at time *t*_0_, learning something about the state of the system *inside R* at time *t*_0_ gives us *no further information* about the eventual state inside *R′* at the later time *t*.^59^

We now define the *distance d*(*R*, *R′*) between *R* and *R′* to be the *maximum* time *t* in *T* such that *R′* is not reachable from *R* in time *t*, if this maximum exists.^60^ This can be interpreted as the *minimum time* required for information to “propagate” from *R* to *R′*. It would be natural to suppose that this notion of distance satisfies three properties:

**Symmetry:** For all subsets *R*, *R′* of *S*,

$$ d(R,R^{{\prime }} ) = d(R^{{\prime }} ,R). $$

**Triangle inequality:** For all subsets *R*, *R′*, *R″* of *S*,

$$ d(R,R^{{\prime \prime }} ) \le d(R,R^{{\prime }} ) + d(R^{{\prime }} ,R^{{\prime \prime }} ). $$

**Non-complementarity:** For all subsets *R*_1_, *R*_2_, *R*_3_ of *S*,

$$ d(R_{1} \cup R_{2} ,R_{3} ) = \hbox{min} \{ d(R_{1} ,R_{3} ),d(R_{2} ,R_{3} )\} . $$

However, none of these properties can be guaranteed, unless the conditional probability structure {*Pr*_*E*_}_*E*⊆Ω_ has the right underlying properties. For example, if the information flow between different spatial locations is asymmetrical, such as in many communications networks, then Symmetry might not be satisfied; it might take longer for information to propagate from *R* to *R′* than vice versa. If information can be “forgotten” or “erased” at some spatial locations in the system, then the Triangle Inequality might not be satisfied; some information propagating from *R* to *R′* might be forgotten before it reaches *R″*. Turning to Non-Complementarity: it is always true that $d(R_{1} \, \cup \,R_{2} ,R_{3} )\, \le \,\hbox{min} \left\{ {d(R_{1} ,R_{3} ),d(R_{2} ,R_{3} )} \right\}$. However, this inequality could be strict; i.e., we could have $d(R_{1} \, \cup \,R_{2} ,R_{3} )\, < \,\hbox{min} \left\{ {d(R_{1} ,R_{3} ),d(R_{2} ,R_{3} )} \right\}$. For example, what happens in regions *R*_1_ and *R*_2_ at time *t*_1_ could be like two pieces of a puzzle, which reveal little about what happens in region *R*_3_ at time *t*_2_ when considered separately, but determine it completely when put together.^61^

Note that our definition of distance between *regions* of space immediately entails a definition of distance between *points* in space: the distance between any two points *s*_1_ and *s*_2_ in *S* is simply the distance between the singleton regions consisting of them, i.e., *d*(*s*_1_, *s*_2_) = *d*({*s*_1_},{*s*_2_}). Clearly, *d*(*s*, *s*) = 0 for any point *s* in *S*. Thus, if our distance measure satisfies Symmetry and the Triangle Inequality, it determines a *metric* on the space *S* (or a *pseudo*-*metric* if *d*(*s*_1_, *s*_2_) = 0 for some *s*_1_ ≠ *s*_2_). Furthermore, if it satisfies Non-Complementarity, this metric completely determines the distance between any two regions *R* and *R′* in *S*.^62^ However, as we have pointed out, the distance measure need not generally satisfy these properties.

One notable feature of the present approach is that it measures the distance between spatial locations in units of *time*. This is, of course, consistent with the practice in modern physics of measuring distance in units such as *light seconds* or *light years*. However, the approach works only if the maximum speed of information propagation in our system is finite. In classical physics, information can propagate through space at arbitrarily high speeds. Therefore, in a classical physical system, the effective “distance” between any two spatial locations collapses to zero, according to our definition. To recover a non-trivial definition of “distance” in such a system, we must impose some restriction on the sort of “information transmission” we can use. For instance, we could consider information transmission via some messenger or signal travelling at a fixed velocity. Similarly, in Maxwell’s theory of electrodynamics, which is complementary to classical mechanics, electromagnetic waves propagate at a fixed and finite speed, namely the speed of light, even if classical-mechanical particles can exceed this speed. Thus, in the world of classical physics, we could define a non-trivial concept of “electromagnetic distance”, even if there is no non-trivial concept of “mechanical distance”. We discuss the issue of distance in quantum mechanics in “Appendix D”.

## Amorphous systems: space–time as an emergent property

### Basic definitions

So far, we have defined histories as functions from a set of points in time or space–time into some state space, where histories play the role of possible worlds. Time or space–time, in turn, had an exogenously given structure. In a temporally evolving system, time was some linearly ordered set (*T*), and in a spatially extended system, space–time was explicitly decomposed into space (*S*) and time (*T*), consistent with some fixed geometry. This picture can, and for many purposes must, be generalized. Both special and general relativity theory, for example, go against the idea that there exists a fixed temporal dimension (for a classic discussion, see Putnam 1967).

A more general approach is to define a history as a function from some “index set”, which we call a *set of loci*, into a state space. The set of loci could be a linearly ordered set of points in time, thereby accommodating temporally evolving systems, or a set of space–time locations with an explicit decomposition into space and time, thereby accommodating spatially extended systems. But it could also be a more general four-dimensional space–time manifold without any exogenous decomposition, or even a completely abstract index set.

Formally, let *I* (for “index set”) be the *set of loci*, and let *X* denote the *state space*. A *generalized history* is a function *h* from *I* into *X*, where, for each locus *i* in *I*, *h*(*i*) is the state of the system at locus *i*. As in the case of spatially extended systems, the state *h*(*i*) is best interpreted, not as a “global” state in which the system is at some specific point in time (indeed, there is no exogenous notion of time), but as a “local” state in which the system is at a specific locus. We write Ω to denote the set of all generalized histories deemed *possible*, which can again be viewed as nomologically possible worlds, and subsets of Ω are called *events*.^63^

To complete the definition of what we call an *amorphous system*, we must, once more, introduce a *conditional probability structure* on Ω. As should be clear by now, this is a family of conditional probability functions {*Pr*_*E*_}_*E*⊆Ω_, consisting of one *Pr*_*E*_ for each event *E* in Ω. Now an *amorphous system* is the pair consisting of the set Ω of nomologically possible generalized histories and the conditional probability structure {*Pr*_*E*_}_*E*⊆Ω_.

How much of our earlier framework can be extended to amorphous systems? We might ask, for instance, whether an abstract index set, despite not being endowed with any exogenous structure, can attain some spatial and/or temporal structure as an emergent property, for instance as a byproduct of the correlations encoded in {*Pr*_*E*_}_*E*⊆Ω_. We might also ask whether, and to what extent, the geometry of the set of loci is unique, or whether there might be multiple, equally admissible geometries.

### Adjacency structure and the Markov property

Just as in Sect. 3.8, the topology of the set *I* of loci can be represented by an *adjacency structure*: a binary relation → defined between subsets of *I*. For example, suppose *I* is a set of times, as in Sect. 2, i.e., *I* = *T*. For any subsets *R* and *R′* of *I*, define *R* → *R′* if there does not exist any time *t* such that *r* < *t* < *r′* for all *r* in *R* and all *r′* in *R′*. For another example, let *I* be the four-dimensional space–time manifold of a general relativistic system. Then, for any subsets *R* and *R′* of *I*, we might define *R* → *R′* if there is a locus *i* in *R* such that every open neighbourhood around *i* intersects *R′*.

In Sect. 2.9, we related the order structure of the set *T* of times to the conditional probability structure {*Pr*_*E*_}_*E*⊆Ω_ by means of a temporal Markov property. Likewise, in Sect. 3.8, we related the adjacency structure of the set *S* of spatial locations to the conditional probability structure {*Pr*_*E*_}_*E*⊆Ω_ by means of a spatial Markov property. We now discuss a similar idea concerning a general set of loci. This will allow us to view the adjacency structure among loci, and thereby its topology, as an “emergent property”: something that emerges from the correlations between events encoded in {*Pr*_*E*_}_*E*⊆Ω_.

Let *R* be a subset of *I* (i.e., a collection of loci). As before, for any generalized history *h* in Ω, we define *h*_*R*_ to be the restriction of that history to the set *R*. We then define the event [*h*_*R*_] to be the set of all histories *h′* in Ω such that $h_{R}  = h_{R}^{\prime}$. For any event *E* (i.e., a subset of Ω), we say that *E happens inside R* if, for all histories *h* and *h′* such that $h_{R}  = h_{R}^{\prime}$, history *h* is an element of *E* if and only if history *h′* is an element of *E.* That is, whether or not a particular history is an element of *E* is completely determined by the restriction of that history to *R*.

As in Sect. 3.8, we define a *tripartition* of the set *I* of loci as a triple (*R*, *R′*, *R″*), where *R*, *R′*, and *R″* are disjoint subsets of *I* which together cover *I* (i.e., *R* ∪ *R′* ∪ *R″* = *I*), such that it is *not* the case that *R* → *R″* or *R″* → *R*. Again, this means that the set *R′* “separates” *R* from *R″*.

For example, let *I* be a set of times (*I* = *T*) with the adjacency structure introduced at the start of this section. Fix two times *t*_0_ and *t*_1_ with *t*_0_ ≤ *t*_1_. Let *R* be the set of all times *strictly before t*_0_, let *R′* be the set of all times *between t*_0_ and *t*_1_ (including *t*_0_ and *t*_1_), and let *R″* be the set of all times *strictly after t*_1_. Then (*R*, *R′*, *R′’*) is a tripartition of *I*.

For another example, let *I* be the four-dimensional Minkowski space–time of special relativity, with the “open neighbourhood” adjacency structure introduced above. Let λ be a linear time-like trajectory through *I*, for instance the trajectory of an “observer” traveling through space–time at a constant velocity, and let *p* be a point on this trajectory. In special relativity theory, there is a unique three-dimensional *simultaneity hyperplane R′* passing through *p*, such that all events that happen inside *R′* seem to occur simultaneously from the perspective of the λ-observer at *p*. Let *R* be the set of all points in *I* which have some part of *R′* in their *future* light-cone, and let *R″* be the set of all points in *I* which have some part of *R′* in their *past* light-cone. Then $(R,R^{{\prime }} ,R^{{\prime \prime }} )$ is a tripartition of *I*.^64^ More generally, let *R* and *R″* be any disjoint open subsets of *I*,^65^ and let *R′* be the complement of the union *R* ∪ *R″.* Then $(R,R^{{\prime }} ,R^{{\prime \prime }} )$ is a tripartition of *I.*

We say that the adjacency structure → satisfies the *amorphous* *Markov property* with respect to the conditional probability structure {*Pr*_*E*_}_*E*⊆Ω_ if, for any tripartition (*R*, *R′*, *R″*) and any generalized history *h* in Ω, any event which happens inside *R* is conditionally independent from any event which happens inside *R″*, given [*h*_*R′*_]. Again, this means, roughly, that there is no way for information to propagate from *R* into *R″*, or vice versa, without first passing through *R′*. For example, suppose *I* is four-dimensional Minkowski space–time, and $(R,R^{{\prime }} ,R^{{\prime \prime }} )$ is the tripartition described above. In this case, the simultaneity hyperplane *R′* plays the role of the “present”, which isolates the “past” *R* from the “future” *R″*. If we have complete information about the history inside *R′* (i.e., we know [*h*_*R′*_]), then we have complete information about the “present state” of the world. Thus, we can predict its future evolution (in *R″*) without needing to know anything about its past history (in *R*).

In Sect. 2.9, we argued that the temporal Markov property was the key property of time; a “correct” ordering of the set *T* was any ordering that satisfied this property. Likewise, in Sect. 3.8, we argued that the spatial Markov property was the key property of space; a “correct” adjacency structure on the set *S* was any adjacency structure that satisfied this property. Now we make a parallel claim for amorphous systems: a “correct” adjacency structure on *I* is one that satisfies the amorphous Markov property. This Markov property subsumes both the temporal Markov property of Sect. 2 and the spatial Markov property of Sect. 3.

This has an important consequence. The topology of the index set *I*, in the form of the adjacency structure, does not need to be imposed exogenously. Instead, this topology can emerge *endogenously* from the conditional probability structure {*Pr*_*E*_}_*E*⊆Ω_. We say that an adjacency structure → between subsets of *I* is {*Pr*_*E*_}_*E*⊆Ω_-*admissible* if it satisfies the amorphous Markov property with respect to {*Pr*_*E*_}_*E*⊆Ω_. If we think of *I* as a sort of generalized space–time, this means that the topology of space–time is an emergent property of the amorphous system.^66^

### Time and predictability

Both temporally evolving systems and spatially extended systems come with a set *T* which plays the role of “time”. What plays the role of time in an amorphous system? The adjacency structure described in the previous section tells us whether two subsets of the index set *I* are in “informational contact” or are “informationally separated” from one another, but it does not tell us which subset comes “before” and which comes “after”, or even whether this question makes sense. We now explain how time itself can be an emergent property of an amorphous system.

Let → be an adjacency structure on the index set *I*. Let *T* be a linearly ordered set. A *possible time structure* on *I* is a function τ from *I* onto *T* (i.e., with *T* = τ(*I*)) such that, for any *t* in *T*, if (i) *R* is the set of all points *i* in *I* such that τ(*i*) < *t*, (ii) *R′* is the set of all points *i* in *I* such that τ(*i*) = *t*, and (iii) *R″* is the set of all points *i* in *I* such that τ(*i*) > *t*, then (*R*, *R′*, *R″*) is a tripartition of *I*. Heuristically, the function τ specifies, for each locus in *I*, the time at which that locus occurs, according to the given time structure.

For example, let *I* be four-dimensional Minkowski space–time as described in Sect. 4.2, and let λ be a linear time-like trajectory through *I.* Fix some point *p*_0_ on the trajectory λ. Let *T* be the set of real numbers. Then, for every *t* in *T*, there is a unique point *p*_*t*_ along the trajectory λ which appears to be *t* seconds in the future of *p*_0_ (or in the past, if *t* < 0), with respect to the subjective time (i.e., *proper time*) experienced by an observer traveling along the trajectory λ. Let *R*_*t*_ be the simultaneity hyperplane passing through *p*_*t*_. If we define τ(*i*) = *t* for all points *i* in *R*_*t*_, then τ is a possible time structure on *I*.

As this example illustrates, an amorphous system may admit *many* possible time structures. In special relativity, there is a distinct time-structure for every inertial reference frame. All of these time structures are equally “correct”. Indeed, this is one of the key insights of special relativity theory. However, unless we impose further constraints, a system may also admit many “absurd” time structures. For example, suppose *I* is four-dimensional Newtonian space–time (i.e., *I* = **R**^3^ × **R**), with the “open neighbourhood” adjacency structure described in Sect. 4.2. For all points $(s_{1} ,s_{2} ,s_{3} ,t)$ in *I*, define $\uptau(s_{1} ,s_{2} ,s_{3} ,t) = s_{3}$. Then τ is a possible time structure on *I*. But if the “true” time coordinate is *t*, not *s*_3_, it seems that this time structure is not correct. So, what property of the system determines which time structures are the correct ones?

Clearly, a “correct” time structure should satisfy something like the temporal Markov property from Sect. 2. However, if the adjacency structure → satisfies the amorphous Markov property with respect to the conditional probability structure {*Pr*_*E*_}_*E*⊆Ω_, then it is easy to see that *any* possible time structure will satisfy the temporal Markov property.^67^ So, the Markov property alone is not enough to pick out the “correct” time structures.

Arguably, what picks out the correct time structures is *predictability*. To understand this, suppose we took a classical mechanical system with Newtonian space–time *I* = **R**^3^ × **R**, and applied the “absurd” time structure $\uptau(s_{1} ,s_{2} ,s_{3} ,t) = s_{3}$, as defined above. How would the system appear with respect to this time structure? It would appear very strange and unpredictable. Particles would randomly pop in and out of existence. Energy and momentum would not be conserved from one moment to the next. Events would seem to unfold over time without any rhyme or reason. This total lack of predictability would be an indication that we had picked the wrong time structure for the system.

On the other hand, if we had picked the “correct” time structure, namely $\uptau(s_{1} ,s_{2} ,s_{3} ,t) = t$, then the system would appear completely deterministic; its state at one “moment” in time, as defined by τ, would completely determine its “past” and “future” behaviour, as defined by τ. This total predictability is an indication that this is the correct time structure for the system.

In this example, there was a particularly stark contrast between an “incorrect” time structure, which renders the system totally unpredictable, and a “correct” one, which renders it totally predictable. This is because classical mechanical systems are deterministic. In an indeterministic system, there will not generally be such a stark contrast. Nevertheless, some time structures will render the system *more* predictable than others, and among these, we claim, the ones that render the system *most predictable* are the correct time structures for that system.

To make this idea more precise, we need a way to measure the “predictability” of a system under a given time structure. One way to do this is to use the information-theoretic notion of *entropy*.^68^ For any subset *R* of *I*, let Ω_*R*_ be the set of all *R*-restricted histories *h*_*R*_ obtained from any *h* in Ω. For simplicity, let us assume that the underlying state space *X* is finite. If *R′* is some other finite subset of *I*, then Ω_*R′*_ is also finite.^69^ Suppose we know *h*_*R*_, and we want to predict *h*_*R′*_. For any *h*_*R*_ in Ω_*R*_, there is a quantity called the *conditional entropy of R′ given h*_*R*_, denoted by η(*R′*, *h*_*R*_), which measures how “unpredictable” the restricted history *h*_*R′*_ is, given the restricted history *h*_*R*_.^70^ For example, if *h*_*R′*_ is entirely determined by *h*_*R*_, then η(*R′*, *h*_*R*_) = 0. At the other extreme, if *h*_*R′*_ is effectively as unpredictable as a collection of independent coin-tosses, even after conditioning on *h*_*R*_, then η(*R′*, *h*_*R*_) = 1. Intermediate levels of entropy represent intermediate degrees of unpredictability.

Now, let τ be a time structure, mapping *I* into *T*. Let *t* be some time in *T*; let *R* be the set of all points *i* in *I* such that τ(*i*) = *t*; and let *R*^C^ be the set of all points *i* in *I* such that τ(*i*) ≠ *t*. We define η(τ, *t*), the *unpredictability* of the system under τ at *t*, to be the maximum value of η(*R′*, *h*_*R*_), where *h*_*R*_ can be any element of Ω_*R*_ and *R′* is allowed to be any finite subset of *R*^C^.^71^ If η(τ, *t*) = 0, then this means roughly that any generalized history *h* in Ω is almost entirely predictable, based on its restriction *h*_*R*_.^72^ If η(τ, *t*) > 0, then histories in Ω are not, in general, fully predictable from their restrictions to *R*. The larger η(τ, *t*) is, the less predictable these histories are. We then define η(τ), the *unpredictability* of the system under the time structure τ, to be the maximum value of η(τ, *t*) over all times *t* in *T*.^73^

For example, suppose *I* is the four-dimensional Newtonian space–time of a classical mechanical system (i.e., *I* = **R**^3^ × **R**), and τ is the “correct” time structure for this system, namely τ(*s*_1_, *s*_2_, *s*_3_, *t*) = *t*. Then η(τ) = 0, because classical mechanics is entirely deterministic. However, if τ was an “incorrect” time structure, such as τ(*s*_1_, *s*_2_, *s*_3_, *t*) = *s*_3_, then we would have η(τ) > 0, because the ascription of this incorrect time structure would render the system unpredictable, as we have explained.

We now come to the key point of this section. A *correct* time structure for an amorphous system is one that *minimizes* unpredictability and thereby maximizes regularity. This definition allows that there may be many correct time structures, all of which render the system equally predictable, as in the case in special or general relativity. This has an important consequence. The correct time structure does not need to be imposed exogenously. Instead, the correct time structure (or structures) could emerge *endogenously* from the conditional probability structure {*Pr*_*E*_}_*E*⊆Ω_. In other words, the structure of time itself could be an emergent property of the amorphous system. Using a more metaphysical language, it might be that space and time are grounded in the dynamics of the system, rather than the other way around.

### Which features of a system are real?

A final philosophical question on which we wish to comment briefly is the following. Suppose we have described a given system using our formal framework. Should we treat all features of that system as “real”, or should we treat some features as mere artefacts of our formal description?

The debates between *relational* and *substantival* views about space and time, and between *structuralist* and *full*-*blown realist* views in science more generally, can be seen as attempts to answer this question.^74^ Let us begin with a *relational* or *structuralist* view, which may be about space and time in particular or about the properties of a system more generally. On such a view (of which there can be several variants), only some “relational” or “structural” properties of a system count as real, while “intrinsic”, “non-structural” properties do not. It does not matter, for instance, what the nature of the system’s spatiotemporal loci in the set *I* is, nor what the nature of the system’s possible states in the set *X* is. All that matters is how these loci and/or states are related to one another and what dynamics they display. Two formally distinct systems, with formally distinct index sets *I* and *I′* and/or formally distinct state spaces *X* and *X′*, will count as the same if their nomologically possible histories and conditional probability structures are structurally indistinguishable.

By contrast, on a *substantival* or *full*-*blown realist* view, which may also be about space and time in particular or about the properties of a system more generally, even intrinsic, non-structural properties of a system can be real, over and above the system’s relational or structural properties. So, the system’s spatiotemporal index set *I* and its state space *X* may be significant in ways that go beyond the structures and relations in which they stand. (Again, there can be several variants of such a view.) An example of a non-structural property is the exact index of time. One can imagine two structurally identical temporally evolving systems, indexed by *T* = {0, 1, 2, 3,…} and *T′* = {1, 2, 3, 4,…}, respectively. The only difference is that in one system history “starts at time zero”, whereas in the other it “starts at time 1”. For a relationalist or structuralist, these are “the same” system. But a substantivalist or full-blown realist might insist that there is a genuine difference between them.

The debates between these different views occur in several places in philosophy and take a variety of forms, so we cannot do justice to them here. We wish to note, however, that our formal framework can be used to express some salient positions within those debates. Specifically, different answers to the question of which features of a system are real can be expressed in terms of different criteria for individuating systems. If we begin with a very large class of systems that are formally described in our framework, there are a number of ways in which one might partition this class of systems into equivalence classes that are each taken to represent the same system. Different such partitions then correspond to different answers to the question of which features of a system are real, rather than mere artefacts of our formal description. In particular, only those features that are present among *all* members of any given equivalence class count as real. Features on which there can be differences even *within* the same equivalence class count as artefacts of our formal description.

A relational or structuralist view would entail that any two systems that do not differ in any relational or structural properties count as the same and thereby fall into the same equivalence class. A substantival or full-blown realist view, by contrast, would entail that two such systems could still count as different; thus, the equivalence classes would be more fine-grained according to such a view, and might even be singleton (in which case *all* features of any given system would count as real).

Here is one way of formalizing this idea. Consider two amorphous systems, given by the pairs $(\Omega ,\{ Pr_{E} \} _{{E \subseteq \Omega }} )\;{\text{and}}\;(\Omega ,\{ Pr_{E}^{{\prime }} \} _{{E \subseteq \Omega ^{{\prime }} }} )$, where the histories in Ω are functions from the set *I* of loci into the state space *X*, and the histories in Ω*′* are functions from the set *I′* of loci into the state space *X′*. Let ${\mathcal{H}}\,{\text{and}}\,{\mathcal{H}}^{\prime}$ denote the sets of logically possible functions from *I* into *X* and from *I′* into *X′*, respectively.

Suppose there is a bijection θ from *I* into *I′*, and also a bijection ξ from *X* into *X′* (recall that a *bijection* is a one-to-one, onto function). Using θ and ξ, we can then define a bijection σ from ${\mathcal{H}}\,{\text{into}}\,{\mathcal{H}}^{\prime}$ which maps each history *h* in ${\mathcal{H}}$ to the history *h′* in ${\mathcal{H}}^{\prime}$ defined as follows: for each *i′* in *I′*,

$$ h^{{\prime }} (i^{{\prime }} ) = \xi [h(i)],\;{\text{where}}\;i =\uptheta^{{{-}1}} (i^{{\prime }} )\;({\text{with}}\;\uptheta^{{{-}1}} \;{\text{defined}}\;{\text{as}}\;{\text{the}}\;{\text{inverse}}\;{\text{of}}\;\uptheta). $$

The bijection σ is an *isomorphism* between the two systems if

- σ(Ω) = Ω*′*; and
- for any events *E′* and *D′* in Ω*′*, if *E* and *D* are the inverse images of *E′* and *D′* under σ, then $Pr^{{\prime }} _{{E^{{\prime }} }} (D^{{\prime }} ) = Pr_{E} (D)$.

We call two systems *isomorphic* if there exists an isomorphism between them. Isomorphic systems display the same dynamics, and they are relationally or structurally indistinguishable.^75^ Moreover, any topology of space and time that is admissible for one such system can be mapped, in a structure-preserving way, onto a topology that is admissible for the other.

Thus, on a relational or structuralist view, any two isomorphic systems should be considered the same. On a substantival or full-blown realist view, they may still differ. A view of the first kind would therefore take systems to be unique only up to isomorphism, so that our initial large class of systems would be partitioned into equivalence classes of isomorphic systems. A view of the second kind would opt for a more fine-grained partition, acknowledging that even isomorphic systems may be distinct in reality.

The properties of systems on which we have focused in this paper are mainly structural and are preserved by all isomorphisms. This includes the symmetries and ergodic properties of a system, the distinction between laws and “brute necessities”, and the topology (or topologies) and geometry (or geometries) of space and time that are compatible with the system’s correlation structure (in the sense that they satisfy the relevant Markov conditions). Thus, even a relationalist or structuralist would accept that all these properties are “real” features of the system, and not mere artefacts.

## Concluding discussion

We have introduced a framework for describing three general classes of systems and shown how it can be used to address a number of philosophical questions. We began with the class of temporally evolving systems, of which classical dynamical systems are a special case, and then moved on to the class of spatially extended systems and the class of amorphous systems. As noted, the framework can accommodate systems as diverse as the solar system, quantum–mechanical systems, special and general relativistic systems, and the earth’s climate system.

We have discussed questions such as: how can we define nomological possibility, necessity, determinism, and indeterminism? What is special about laws, and how are laws related to symmetries? What regularities must a system display to permit global generalizations from local observations? How can we formulate principles of parsimony such as Occam’s Razor? What is the role of space and time in a system? And what is at stake in the debate between relational and substantival views about space and time, and between structuralist and full-blown realist views about systems more generally?

While our framework and what it says about these questions should already be of sufficient interest to make it worth studying, a further payoff lies arguably in the variety of applications to which the framework lends itself. Developing these is beyond the scope of this paper, but we conclude by mentioning a few.

### Higher-level versus lower-level properties

Our framework can be used to explore the relationship between lower-level (“micro”) and higher-level (“macro”) properties of a system. By partitioning the system’s state space *X* into suitable equivalence classes, we can capture the idea that “higher-level” or “macro” states are more coarse-grained than “lower-level” or “micro” states, so that each “macro” state can be realized by different “micro” states: the phenomenon of *multiple realizability*. Consider, for example, all the different possible micro-level trajectories of a tossed coin that each correspond to the macro-property of “landing heads”. Or consider all the different possible micro-states of individual water molecules that each correspond to a macro-state such as “frozen”, “liquid”, or “gaseous”.

Suppose *X* is the original state space, and  is the relevant set of equivalence classes, which we interpret as the higher-level state space. We can then write σ to denote the function that maps each lower-level state *x* in *X* to the corresponding higher-level state  in  . Note the outlined font for higher-level objects. This function can be interpreted as the *supervenience relation* connecting the two levels. We can then use σ to specify the resulting higher-level histories.^76^ For each lower-level history *h* in the original set Ω, the corresponding higher-level history  is the function from *T* into  , where, for each *t* in *T*,  (*t*) = σ(*h*(*t*)). (If we are dealing with a spatially extended or amorphous system instead of a temporally evolving one, we must replace *T* in this definition with *S* × *T* or *I*.) The set of higher-level histories is therefore  = σ(Ω). Similarly, we can use σ to arrive at a conditional probability structure defined over higher-level events, formally written  . See “Appendix A” for details. The pair  can be viewed as our system, *re*-*described at a higher level*. In the terminology of “Appendix A”, the higher-level system is a *factor system* of the original, lower-level system.

This construction allows us to study the dynamics of the higher-level system and to compare its properties with those of the lower-level system. Interestingly, the higher-level dynamics may be different from the underlying lower-level dynamics. For example, features such as determinism or indeterminism are not generally preserved under coarse-graining: the lower-level system may be deterministic, while the higher-level system is not (or vice versa). Thus indeterminism could be an emergent property (see, e.g., Butterfield 2012; List 2014; List and Pivato 2015; and relatedly Werndl 2009b).

In a similar vein, we may study the level-specificity of other properties. For instance, this approach can be used to argue that non-trivial objective chance can be an emergent phenomenon, consistently with lower-level determinism (List and Pivato 2015).^77^

### Laws and regularities in the special sciences

There is much debate on whether there are laws in the special sciences, as distinct from fundamental physics. The existence of laws is particularly contested in fields such as biology, ecology, geology, psychology, and the social sciences. (Chemistry, by contrast, is often viewed as a close relative of physics and thereby similar enough to it in its lawfulness.) Examples of special-science regularities that are sometimes described as laws include (i) Kleiber’s law in biology, according to which an organism’s metabolic rate is proportional to the ¾th power of its body mass; (ii) the laws of supply and demand in economics, according to which (except for Giffen goods) the demand for a good is a decreasing function of its price, and the supply is an increasing function of price; and (iii) Duverger’s law in political science, according to which, under a first-past-the-post electoral system, the effective number of parties in the legislature will be lower than under a proportional-representation system, *ceteris paribus*. The key question is whether any of these regularities are sufficiently robust to qualify as laws.

One common view is that, as we move further away from fundamental physics, there are fewer and fewer regularities that are genuinely law-like. Kim (2010, ch. 14), for instance, argues that there are no “strict” laws in the special sciences. Among the reasons he gives for this conclusion are (i) the multiple realizability of special-science properties, which, he claims, undermines their “inductive projectibility”, and (ii) the alleged metaphysical anomalism of the mental realm, which, he suggests, undermines the existence of laws in psychology and the social sciences.

Other scholars defend the existence of laws in the special sciences. For example, focusing on the social sciences, Kincaid (1990) argues that several widely cited arguments against laws fail. He thinks that the most serious challenge to laws in the social sciences comes from the excessive *ceteris paribus* qualifications that all such laws require, but argues that the procedures we routinely employ to deal with such qualifications in the natural sciences carry over to the social sciences.

Our framework might be used to make some progress in this debate. Using the framework, we can in principle describe the special-science systems in question and identify the properties these systems would have to display in order to secure the existence of laws. Those laws would then have the testable and generalizable character we have discussed. As we have seen, what laws there are in a given system depends on the system’s symmetries and the properties they preserve. This is as true for a system in the special sciences as it is for a physical system. Moreover, our analysis implies that whether, given only local observations, we can gain knowledge of the probabilistic dynamics of a special-science system depends on whether the system is ergodic. The importance of ergodicity for the special sciences is much less widely recognized than its importance for physics.

Interestingly, if a special-science system arises as a higher-level description of a physical system, as discussed in Sect. 5.1, then it will inherit some structure from the physical system, and it will have at least as many temporal or spatiotemporal symmetries as that physical system (and possibly more), and at least as much ergodicity, for reasons explained in “Appendix A”. Another question is whether we are prepared to recognize weaker kinds of laws corresponding to *partial* or *local* symmetries, as defined in “Appendix B”. This question is particularly pertinent for the special sciences, insofar as the systems investigated in fields ranging from biology to the social sciences often have special initial or boundary conditions. While all of these issues are difficult, our framework can help us clarify what is at stake in the debate about special-science laws and thereby render the debate more tractable. For earlier applications of dynamical-systems theory to the special sciences, see Auyang (1998) and Yoshimi (2012).

### Intentional systems

Although there has been no such thing as intentionality in our paradigmatic examples of systems, there is no barrier, in principle, to using our framework also for describing systems involving intentional agents. Indeed, van Gelder (1995) and Juarrero (1999) have argued for understanding cognitive systems as special kinds of dynamical systems (see also Spivey 2008; Hotton and Yoshimi 2010; Silberstein and Chemero 2012); and more recently, a precursor of the present formalism has proved useful for the analysis of free will and agency (List 2014; List and Rabinowicz 2014). We can think of an agent, together with the relevant environment, as a temporally evolving system. This system can be described at different levels: at a physical level, at which we would *not* take an “intentional stance” towards the system, and at an agential level, at which we *would* take such a stance (on the notion of an “intentional stance”, see Dennett 1987). Physical-level descriptions capture the states of the agent’s brain and body, while agential-level descriptions capture the agent’s higher-level mental or psychological states, thereby focusing on the agent’s beliefs, desires, and intentions, rather than the underlying neuronal or bodily states.

The present framework then allows us to explain, for instance, how agential-level indeterminism and an agent’s *possibility of doing otherwise* can co-exist with physical-level determinism (List 2014). The framework might also shed some light on how other psychological properties can emerge from the underlying physical dynamics of the system. In particular, as a factor system of the original physical system, the agential system may exhibit additional symmetries not present at the physical level—a point already alluded to in Sect. 5.2. This may, in turn, be used to explain why some higher-level regularities in an intentional system (e.g., regularities involving beliefs, desires, intentions, and norms) may qualify as “real patterns”, as Dennett (1991) has argued, and not merely as illusions due to our ignorance of the physical-level details.

Needless to say, all of these applications are challenging and raise controversial philosophical issues. We hope, however, that our framework will be a clarifying contribution to formal metaphysics and the philosophy of science and will inspire further work.

## Figures and Tables

**Fig. 1:**
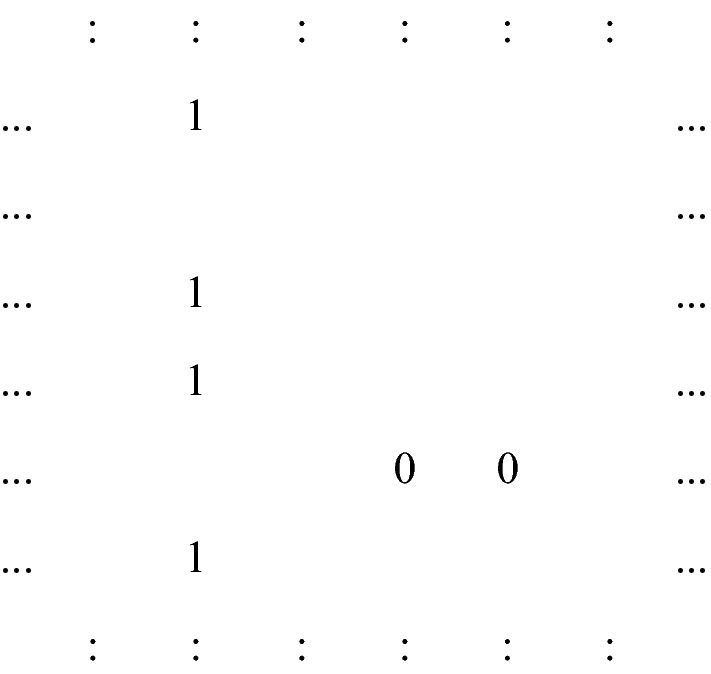
The empirical evidence

**Fig. 2:**
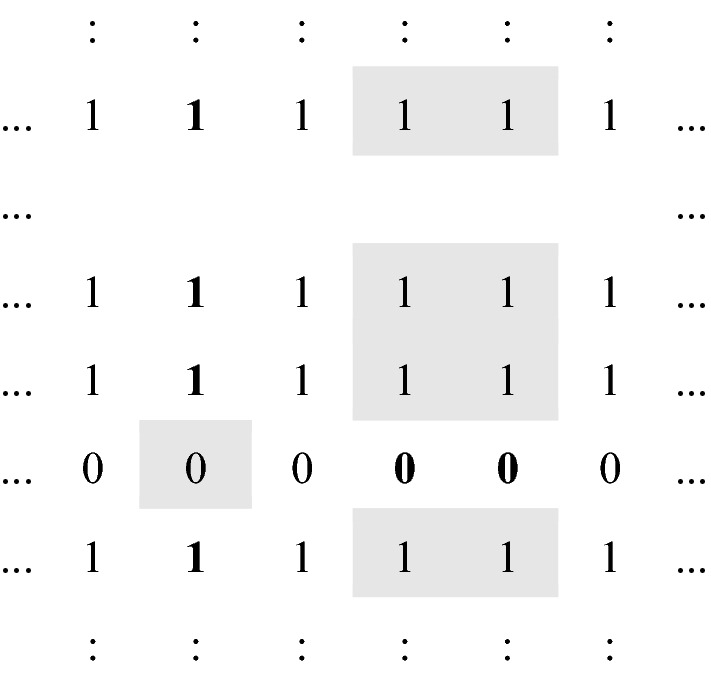
The consequences of Hypothesis 1

**Fig. 3:**
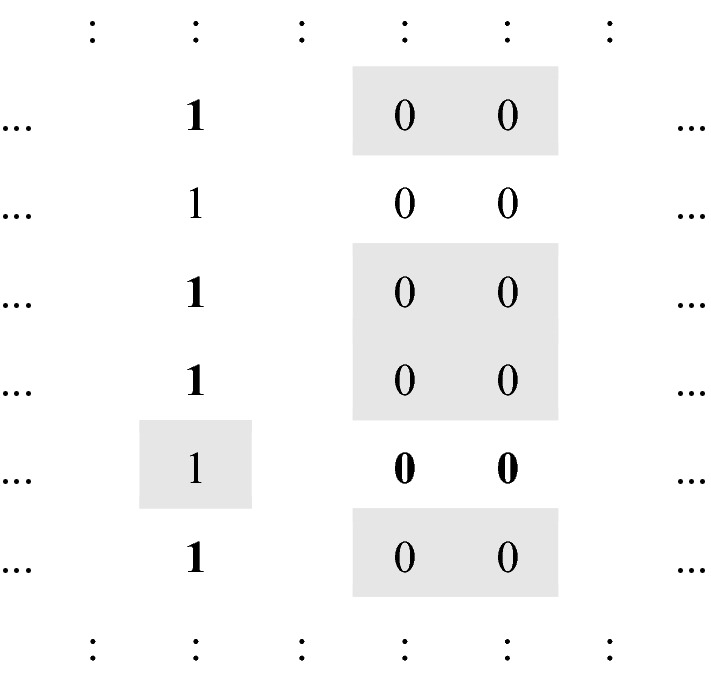
The consequences of Hypothesis 2

**Fig. 4:**
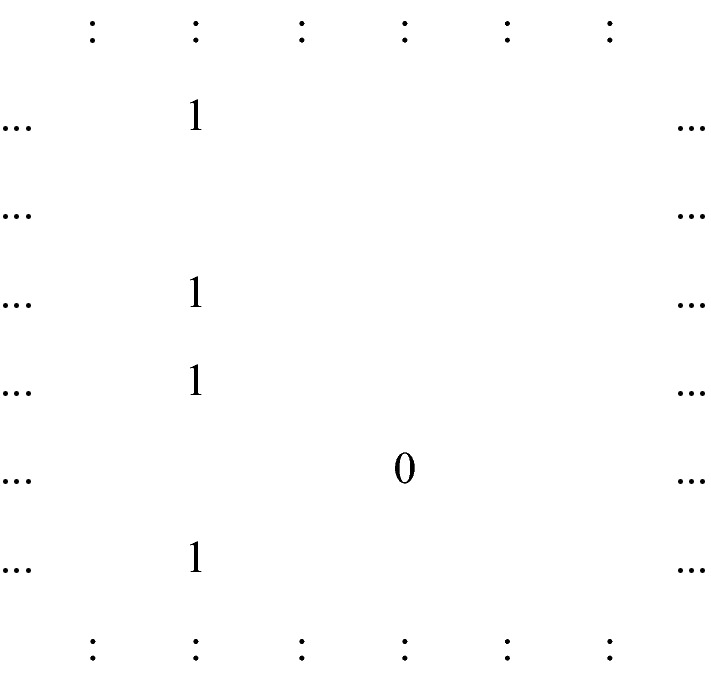
Weaker evidence to obtain Fig. 2 from Hypothesis 1

**Fig. 5:**
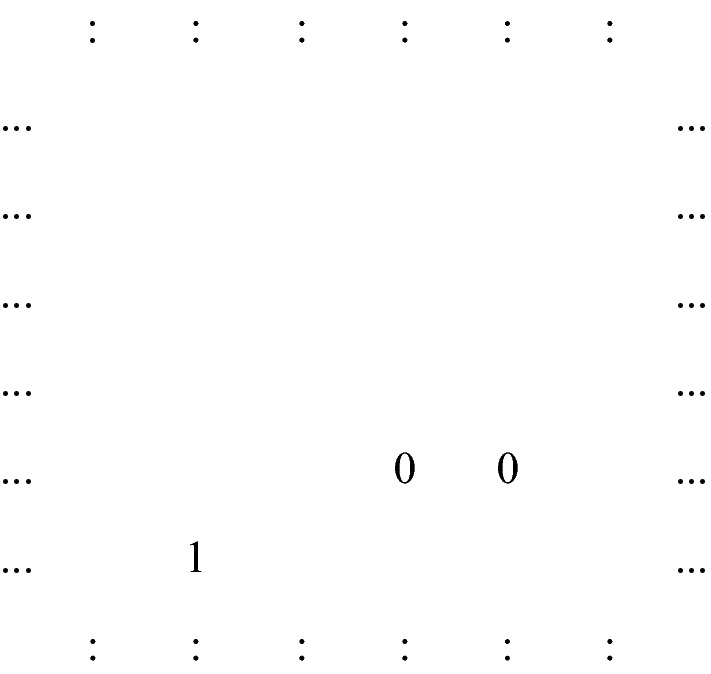
Weaker evidence to obtain Fig. 3 from Hypothesis 2

## Appendix A: Factor systems

One possible objection to our framework is that it is both unrealistic and unwieldy. It is unrealistic because the actual universe is not sufficiently regular (e.g., it might lack a large enough monoid of symmetries or ergodicity). It is unwieldy because the universe as a whole is far too complex a system for us to analyze within this framework anyway.

However, there is no need to insist on applying our framework to the universe as a whole. Instead, we can apply it to a “factor” system, as we now explain. Consider a temporally evolving system, consisting of a set Ω of nomologically possible histories (each of which is a function from a set *T* of times into a set *X* of possible states), along with a conditional probability structure {*Pr*_*E*_}_*E*⊆Ω_. Let *X′* be another set, and let σ be a function from *X* into *X′*. For every history *h* in Ω, let σ(*h*) be the function *h′* from *T* into *X′* defined by *h′*(*t*) = σ(*h*(*t*)) for all *t* in *T*. Let Ω*′* = {σ(*h*): *h* ∈ Ω}. For any subsets *D′* and *E′* of Ω*′*, let *D* and *E* be their inverse images under σ, where these are subsets of Ω, and define $Pr^{{\prime }} _{{E^{{\prime }} }} (D^{{\prime }} ) = Pr_{E} (D)$. Then $\{ Pr^{{\prime }} _{{E^{{\prime }} }} \} _{{E^{{\prime }}  \subseteq \Omega ^{{\prime }} }}$ is a conditional probability structure on Ω*′*. The system specified by Ω′ and $\{ Pr^{{\prime }} _{{E^{{\prime }} }} \} _{{E^{{\prime }}  \subseteq \Omega ^{{\prime }} }}$ is called a *factor system* of the original system specified by Ω and {*Pr*_*E*_}_*E*⊆Ω_. The function σ from Ω into Ω*′* is called a *factor map.* We can define *factors* of spatially extended and amorphous systems analogously.

For a concrete example, suppose that (Ω, {*Pr*_*E*_}_*E*⊆Ω_) is a classical-physics description of the entire solar system at an atomic level of detail. So *X* is an extremely high-dimensional space, which must specify the position and momentum of every atom in the entire solar system, along with all of their gravitational and electromagnetic interactions.^78^ Meanwhile, let $(\Omega ^{{\prime }}, \{ Pr^{{\prime }} _{{E^{{\prime }} }} \} _{{E^{{\prime }}  \subseteq \Omega ^{{\prime }} }} )$ be the very simple celestial-mechanical system consisting only of the Earth, Moon, and Sun, described as gravitationally interacting point masses. So *X′* = **R**^18^, because we must specify the three-dimensional position and momentum vectors of each of the three objects in the system, and 3 × 6 = 18. Let σ be the function from *X* into *X′* which translates each highly detailed atomic-level description of the solar system into the crude 18-dimensional celestial mechanical description. Then σ is a factor mapping from (Ω, {*Pr*_*E*_}_*E*⊆Ω_) into $(\Omega ^{{\prime }}, \{ Pr^{{\prime }} _{{E^{{\prime }} }} \} _{{E^{{\prime }}  \subseteq \Omega ^{{\prime }} }} )$, and thus $(\Omega ^{{\prime }}, \{ Pr^{{\prime }} _{{E^{{\prime }} }} \} _{{E^{{\prime }}  \subseteq \Omega ^{{\prime }} }} )$ is a factor of (Ω, {*Pr*_*E*_}_*E*⊆Ω_).

As this example illustrates, a factor system can be seen as a sort of “abstraction” or “simplification” of the original system, obtained by discarding some properties. Now, suppose ψ is a function from *T* into itself (e.g., a time shift) which is a temporal symmetry of the original system $(\Omega, \{ Pr_{E} \} _{{E \subseteq \Omega }} )$. Then it is easy to verify that ψ will also be a temporal symmetry of the factor system $(\Omega ^{{\prime }}, \{ Pr^{{\prime }} _{{E^{{\prime }} }} \} _{{E^{{\prime }}  \subseteq \Omega ^{{\prime }} }} )$. Thus, the temporal symmetry monoid of the factor system $(\Omega ^{{\prime }}, \{ Pr^{{\prime }} _{{E^{{\prime }} }} \} _{{E^{{\prime }}  \subseteq \Omega ^{{\prime }} }} )$ is *at least as large* as the temporal symmetry monoid of the original system $(\Omega, \{ Pr_{E} \} _{{E \subseteq \Omega }} )$. In a spatially extended system, the exact same statement applies to spatiotemporal symmetries. Furthermore, if Ψ is an amenable monoid of temporal (or spatiotemporal) symmetries, and (Ω, {*Pr*_*E*_}_*E*⊆Ω_) is ergodic relative to Ψ, then $(\Omega^{{\prime }}, \{ Pr^{{\prime }} _{{E^{{\prime }} }} \} _{{E^{{\prime }}  \subseteq \Omega ^{{\prime }} }} )$ will *also* be ergodic relative to Ψ. In other words, $(\Omega ^{{\prime }}, \{ Pr^{{\prime }} _{{E^{{\prime }} }} \} _{{E^{{\prime }}  \subseteq \Omega ^{{\prime }} }} )$ is *at least as ergodic* as $(\Omega,\{ Pr_{E} \} _{{E \subseteq \Omega }} )$.

This means that, even if the original system $(\Omega, \{ Pr_{E} \} _{{E \subseteq \Omega }} )$ lacks certain symmetries or ergodic properties, the factor system $(\Omega ^{{\prime }}, \{ Pr^{{\prime }} _{{E^{{\prime }} }} \} _{{E^{{\prime }}  \subseteq \Omega ^{{\prime }} }} )$ may well possess these properties. Furthermore, even if the original system $(\Omega, \{ Pr_{E} \} _{{E \subseteq \Omega }} )$ is too complicated to analyze using the formal tools we have described, the system $(\Omega ^{{\prime }}, \{ Pr^{{\prime }} _{{E^{{\prime }} }} \} _{{E^{{\prime }}  \subseteq \Omega ^{{\prime }} }} )$ may well be simple enough. To illustrate this, consider our example of the solar system. The original system $(\Omega, \{ Pr_{E} \} _{{E \subseteq \Omega }} )$ describes the entire solar system at an atomic level of detail. Whether or not the system possesses the desired symmetries or ergodic properties, it is certainly too complex to analyze. In contrast, the abstract Earth-Moon-Sun system $(\Omega ^{{\prime }}, \{ Pr^{{\prime }} _{{E^{{\prime }} }} \} _{{E^{{\prime }}  \subseteq \Omega ^{{\prime }} }} )$ is very simple. In fact, it is an example of a *quasiperiodic* dynamical system: it can be described as two independently rotating “wheels”, one describing the orbit of the Moon around the Earth, and the other describing the orbit of the Earth around the Sun. This is a prototypical example of an ergodic dynamical system.

## Appendix B: Partial symmetries and local symmetries

An important assumption of this paper has been that there is a fairly large monoid Ψ of symmetries acting on the set Ω of nomologically possible histories. We have argued that a property of the system qualifies as a “law” only if it is invariant under all of these symmetries. But this argument runs into a problem: many systems studied in the sciences lack sufficient symmetries to account for all of their “law-like” features.

For example, suppose space is represented by the set of all integers, while time is represented by the set of positive integers, i.e., $S = \{  \ldots ,-1,0,1,2, \ldots \}$ and $T = \{ 1,2,3, \ldots \}$, and consider the simple random-walk system described in Sect. 2.9. Nomologically speaking, the token could begin at any spatial location at time one. But suppose the conditional probability structure {*Pr*_*E*_}_*E*⊆Ω_ is such that, with probability one, the token begins at spatial location zero at time one.^79^ In that case, the probability distribution of its location at time *t* is a (*t* − 1, ½)-*binomial distribution*.^80^ Evidently, this distribution is not invariant under spatial translations, since it is centred around zero. Furthermore, it changes over time. Thus, spatiotemporal translations are not symmetries of this system. But this contradicts our intuition that the motion of the token is highly “law-like”: it can be described by a simple rule which is the same everywhere in space and time.

To solve this problem, we now introduce the notion of “partial” symmetries. We use the framework of spatially extended systems. Recall that ${\mathcal{H}}$ is the set all logically possible spatially extended histories. A *partial symmetry monoid* of a spatially extended system is a collection Ψ of transformations of ${\mathcal{H}}$, along with a collection ${\mathcal{E}}$ of ordered pairs of events (*E*, *D*), such that:

- ψ(Ω) = Ω for all ψ in Ψ; and
- for any event pair (*E′*, *D′*) in ${\mathcal{E}}$ and any ψ in Ψ, if *E* and *D* are the inverse images (in Ω) of *E′* and *D′* under ψ, then (*E*, *D*) is also in ${\mathcal{E}}$, and *Pr*_*E*_(*D*) = *Pr*_*E′*_(*D′*).^81^

For example, in our random-walk example (re-construed in the framework of spatially extended systems), let ${\mathcal{E}}$ be the set of all ordered pairs of events (*E*, *D*) such that event *E* exactly specifies the location of the particle at some time *t*, while event *D* happens at some later time *t′*. Thus, *Pr*_*E*_(*D*) is the conditional probability that the token satisfies such-and-such property at time *t′*, *given* that it was at such-and-such location at time *t*. Let Ψ be the monoid of all spatiotemporal translations of *S* × *T*. If ψ is any element of Γ, then the set ${\mathcal{E}}$ of pairs of events is invariant under ψ, and the conditional probability *Pr*_*E*_(*D*) is preserved by ψ for any (*E*, *D*) in ${\mathcal{E}}$, in the sense described above. Thus, the pair (Ψ, ${\mathcal{E}}$) is a partial symmetry monoid for the random-walk system. Crucially, the set ${\mathcal{E}}$ does not include pairs of the form (Ω, *D*), so we do not require unconditional probabilities of the form *Pr*_Ω_(*D*) to be preserved by spatiotemporal translations.

Seen from this perspective, the transition probabilities of the random walk are “law-like”, because they are preserved by all the transformations in Ψ. In contrast, the initial probability distribution of the system is merely a brute necessity of the present system, since it is not preserved by any symmetries.

For another example suppose that the temporally evolving (or spatially extended) system $(\Omega ^{{\prime }}, \{ Pr^{{\prime }} _{{E^{{\prime }} }} \} _{{E^{{\prime }}  \subseteq \Omega ^{{\prime }} }} )$ is a *factor* of the system $(\Omega, \{ Pr_{E} \} _{{E \subseteq \Omega }} )$, via some factor map σ, as described in “Appendix A”. For any event *E′* ⊆ Ω*′*, let σ^−1^(*E′*) denote its inverse image under σ (here defined as a subset of Ω). Then define ${\mathcal{E}}$ = {(σ^−1^(*E′*), σ^−1^(*D′*)): *E′*, *D′* ⊆ Ω*′*}. Let Ψ be a monoid of spatiotemporal symmetries of the factor system $(\Omega ^{{\prime }}, \{ Pr^{{\prime }} _{{E^{{\prime }} }} \} _{{E^{{\prime }}  \subseteq \Omega ^{{\prime }} }} )$. The elements of Ψ might not be symmetries of the original system $(\Omega, \{ Pr_{E} \} _{{E \subseteq \Omega }} )$. However, they will be *partial* symmetries, with respect to the set ${\mathcal{E}}$. So (Ψ, ${\mathcal{E}}$) is a partial symmetry monoid for $(\Omega, \{ Pr_{E} \} _{{E \subseteq \Omega }} )$. As already explained in “Appendix A”, one can greatly extend the scope of our framework by focusing attention on a factor system rather than the original system. We now see that this is a special case of the broader concept of a partial symmetry monoid.

However, partial symmetry monoids cannot accommodate another feature of many systems. To illustrate this, consider a temporally evolving system where time is a *finite* sequence of integers, such as *T* = {1, 2,…,100}. For such a system, time translations are not even *well*-*defined*.^82^ But in most such systems, we still want to say that the system obeys the same causal laws at all times, except perhaps at times 0 and 100. A similar problem arises in a spatially extended system where the space *S* is bounded (e.g., a partial differential equation defined on a cube, with specified boundary conditions) or a finite set of points (e.g., a cellular automaton defined on a 100 × 100 grid, with specified boundary conditions). In such a system, spatial translations are not well-defined. But in most such systems, we still want to say that the system obeys the same causal laws everywhere in the “interior” of the spatial domain.

To solve this problem, we now introduce “local” symmetries. We begin with some preliminary definitions. Let *N* be a subset of *S* × *T*; call this a *neighbourhood* of space–time. Extending our earlier terminology, we say that an event *E* ⊆ Ω *happens inside N* if, for all histories *h* and *h′* in Ω, if $h_{N}  = h_{N}^{\prime}$, then *h*_*N*_ is in *E* if and only if $h_{N}^{\prime}$ is in *E*. Let Ω_*N*_ = {*h*_*N*_: *h* in Ω}; this is the set of all nomologically possible histories restricted to *N* (a set of functions from *N* into *X*). Let *N′* be another neighbourhood of *S* × *T*, and suppose ψ is a function from *N′* into *N.* We use this to construct a function from histories restricted to *N* into histories restricted to *N′*. Specifically, for any *h*_*N*_ in Ω_*N*_, we define ψ(*h*_*N*_) to be the function *h′*_*N′*_ from *N′* into *X* given by $h_{{N^{{\prime }} }}^{\prime } (n^{{\prime }} ) = h_{N} ({\uppsi }(n^{{\prime }}))$, for all *n′* in *N′*. Note that while $h_{{N^{{\prime }} }}^{\prime }$ is a logically possible history restricted to *N′*, it is not necessarily an element of Ω_*N′*_.

We now define a *local symmetry groupoid* of a spatially extended system to be a combination of three components:

- a collection ${\mathcal{N}}$ of subsets of *S* × *T* (called *neighbourhoods*);
- for each neighbourhood *N* in ${\mathcal{N}}$, a set ${\mathcal{E}}_{N}$ of ordered pairs of events (*E*, *D*) which happen inside *N* (called *local events*); and
- for each pair of neighbourhoods *N* and *N′* in ${\mathcal{N}}$, a collection Ψ_*N*,*N′*_ of bijections from *N′* into *N* (called *local symmetries*).

We refer to the collection {Ψ_*N*,*N′*_: *N*, *N′* ∈ ${\mathcal{N}}$} as a *groupoid* because it must satisfy two algebraic closure properties:

- for all neighbourhoods *M* and *N* in ${\mathcal{N}}$, and any local symmetries ψ in Ψ_*M*,*N*_, its inverse ψ^−1^ is in Ψ_*N,M*_; and
- for all neighbourhoods *L*, *N*, and *M* in ${\mathcal{N}}$, and all local symmetries α in Ψ_*L*,*M*_ and β in Ψ_*M*,*N*_, the composition α ο β is in Ψ_*L,N*_.

For any neighbourhoods *N* and *N′* in ${\mathcal{N}}$, and any ψ in Ψ_*N,N′*_, we call ψ a *local symmetry* because it must preserve the modal and probabilistic structure of the system in the following sense:

- ψ(Ω_*N*_) = Ω_*N′*_; and
- for all event pairs (*E′*, *D′*) in ${\mathcal{E}}_{{N}^{\prime}}$, if *E* and *D* are the inverse images (in Ω_*N*_) of *E′* and *D′* under ψ, then (*E*, *D*) is in ${\mathcal{E}}_{N}$, and *Pr*_*E*_(*D*) = *Pr*_*E′*_(*D′)*.^83^

For example, suppose that *S* = {1, 2,…,10} and *T* = {1, 2,…,100}. For any *s* in {2,…,9} and *t* in {2,…,99}, let *N*_*s,t*_ be the 3 × 2 “space–time rectangle” of the form $N_{{s,t}}  = \{ s - 1,s,s + 1\}  \times \{ t,t + 1\}$. Let ${\mathcal{N}}$ be the set of all such space–time rectangles. For any *s* and *s′* in {2,…,9}, and any *t* and *t′* in {2,…,99}, if *N* = *N*_*s,t*_ and $N^{{\prime }}  = N_{{s^{{\prime }} ,t^{{\prime }} }}$, then we define $\Psi _{{N,N^{{\prime }} }}  = \{ \uppsi _{{s^{{\prime }} ,t^{{\prime }}  \to s,t}} \}$, where $\uppsi _{{s^{{\prime }} ,t^{{\prime }}  \to s,t}}$ is the function from *N′* into *N* which sends each space–time point (*s*_0_, *t*_0_) in *N′* to the point $(s_{0}  - s^{{\prime }}  + s,t_{0}  - t^{{\prime }}  + t)\;{\text{in}}\;N$. Then, with a suitable specification of the local event sets ${\mathcal{E}}_{N}$ for all *N* in ${\mathcal{N}}$, we could construct a local symmetry groupoid for many of the spatially extended systems (such as cellular automata) that one might define on *S* × *T.* However, a fully worked out example would be rather technically involved and is beyond the scope of this paper; see Golubitsky et al. (2003) and Guay and Hepburn (2009).

Most of the ideas we have developed in this paper for the monoid of “full” symmetries can be generalized to partial symmetries and local symmetries. However, this is also beyond the scope of this paper.

## Appendix C: Inferential modesty, informational parsimony, and the nomological hypothesis

Our version of Occam’s Razor requires us to assume that the symmetries of our system are given by a maximal symmetry monoid consistent with our total nomological evidence *E* (a superset of Ω). Under natural assumptions, at least one maximal consistent monoid will indeed exist.^84^ However, there may be more than one. In this case, we need a criterion to choose one maximal symmetry monoid rather than another. We now develop such a criterion.

Let us begin with an example. Consider a very simple temporally evolving system, where the set *T* of times contains only a single element. So, histories can be identified with states at that single time; this expositional simplification has no substantive consequences. Suppose that the state of the system is described by a two-dimensional grid of zeros and ones, which is infinite in every direction. Let *X* be the set of all logically possible grids of this kind. Then the set ${\mathcal{H}}$ of all logically possible histories can be identified with *X*. In this system, one elementary kind of nomological constraint is one that constrains the values of one or more cells, for example the constraint “in any possible history, the cell (2, 3) must have the value zero”.^85^ Suppose we have obtained evidence that any possible history must satisfy the constraints shown in Fig. 1. This evidence would be represented by the subset *E* of ${\mathcal{H}}$ consisting of all single-period histories in which the grid coincides with Fig. 1 in all non-empty cells. For the sake of argument, let us treat *E* as our total nomological evidence about the system.

Now, for any integer *n*, let ψ_*n*_^→^ be the transformation that shifts the entire grid to the *right* by *n* spaces.^86^ Let $\Psi^{ \to } := \{  \ldots ,\uppsi _{{{-}1}} ^{ \to } ,\uppsi _{0} ^{ \to } ,\uppsi _{1} ^{ \to } ,\uppsi _{2} ^{ \to } ,...\}$ denote the monoid of all such horizontal shifts. Meanwhile, let ψ_*n*_^↑^ be the transformation that shifts the entire grid *upwards* by *n* spaces, and let $\Psi^{ \uparrow } := \{  \ldots ,\uppsi _{{{-}1}} ^{ \uparrow } ,\uppsi _{0} ^{ \uparrow } ,\uppsi _{1} ^{ \uparrow } ,\uppsi _{2} ^{ \uparrow } ,...\}$ denote the monoid of all such vertical shifts. Consider two hypotheses: **Hypothesis 1:** All transformations in Ψ^→^ are symmetries of the system. **Hypothesis 2:** All transformations in Ψ^↑^ are symmetries of the system.

The evidence represented in Fig. 1 is consistent with either of these hypotheses. However, it cannot accommodate both simultaneously. If Hypothesis 1 were true, then the constraints in Fig. 1 would entail the constraints shown in Fig. 2. If Hypothesis 2 were true, then they would entail the constraints shown in Fig. 3. In each figure, the constraints that were part of the initial nomological evidence are highlighted in boldface; extrapolated constraints (based on the postulated symmetries) appear in non-bold font. Clearly, Hypotheses 1 and 2 cannot *both* be true, since they yield mutually contradictory constraints on the values of the grey cells.

Let Ψ be some maximal consistent monoid of transformations that we postulate as the symmetry monoid, in accordance with Occam’s Razor. Hypothesis 1 then asserts that Ψ^→^ ⊆ Ψ, while Hypothesis 2 asserts that Ψ^↑^ ⊆ Ψ. Since both hypotheses cannot simultaneously be true, it follows that there are at least two distinct ways in which we could specify Ψ: one including Ψ^→^ and another including Ψ^↑^. So even in this very simple example, there is no unique maximal consistent monoid.

At first sight, the choice between these two maximal symmetry monoids seems arbitrary. But it is not. To see this, note that both hypotheses could have entailed the same constraints they did, using less initial evidence. For example, Hypothesis 1 would have entailed the same constraints from the weaker evidence represented in Fig. 4.

The original evidence in Fig. 1 constrained *six* cell values (i.e., six “bits” of information). But Hypothesis 1 can make do with only *five* of them (in particular, the second zero is redundant). Meanwhile, Hypothesis 2 would have entailed the same constraints from only *three* bits of information, as represented in Fig. 5.

In other words, Hypothesis 2 could have entailed all of its original constraints, using *less information* than Hypotheses 1 needed to obtain its original constraints. Thus Hypothesis 2 can be viewed as more *informationally parsimonious* than Hypothesis 1. Hypothesis 2 stands out in another way too: from the same initial evidence, it constrains *fewer cell values* than Hypothesis 1. So, Hypothesis 2 is also more *inferentially modest* than Hypothesis 1.

This simple example illustrates two general points. First, different symmetry monoids may lead to different nomological hypotheses—hypotheses about what the nomologically possible histories are—even starting from the same body of nomological evidence. Formally, we may have Ω(Ψ_1_, *E*) ≠ Ω(Ψ_2_, *E*) where Ψ_1_ and Ψ_2_ are two distinct symmetry monoids that are each consistent with *E*. Second, one symmetry monoid could generate the *same* nomological hypothesis from two different bodies of nomological evidence. Formally, we may have Ω(Ψ, *E*_1_) = Ω(Ψ, *E*_2_) for the same symmetry monoid Ψ and two distinct sets *E*_1_ and *E*_2_.

Thus, given two symmetry monoids Ψ_1_ and Ψ_2_, which are each compatible with the same total nomological evidence *E*, we can compare them along two dimensions: **Inferential modesty:** If Ω(Ψ_2_, *E*) ⊆ Ω(Ψ_1_, *E*), then we say that Ψ_1_ is (at least weakly) more *inferentially modest* than Ψ_2_. **Informational parsimony:** Let *E*_1_ be the largest superset^87^ of *E* such that Ω(Ψ_1_, *E*_1_) = Ω(Ψ_1_, *E*). Let *E*_2_ be the largest superset of *E* such that Ω(Ψ_2_, *E*_2_) = Ω(Ψ_2_, *E*). If *E*_2_ ⊆ *E*_1_, then we say that Ψ_1_ is (at least weakly) more *informationally parsimonious* than Ψ_2_.

Returning to our earlier example with the infinite grid, let *E* be the nomological evidence described by Fig. 1. Then Ω(Ψ^→^, *E*) is the set of single-period histories satisfying the constraints described by Fig. 2, and Ω(Ψ^↑^, *E*) is the corresponding set for Fig. 3. Meanwhile, if *E*^→^ is the nomological evidence described by Fig. 4, then we have $\Omega (\Psi ^{ \to } ,E^{ \to } ) = \Omega (\Psi ^{ \to } ,E)$. Likewise, if *E*^↑^ is the nomological evidence described by Fig. 5, then we have $\Omega (\Psi ^{ \uparrow } ,E^{ \uparrow  } ) = \Omega (\Psi ^{ \uparrow } ,E)$.

In this example, neither Ω(Ψ^→^, *E*) nor Ω(Ψ^↑^, *E*) includes the other, so neither monoid is more inferentially modest than the other, according to our definition. Likewise, neither *E*^→^ nor *E*^↑^ includes the other, so neither monoid is more informationally parsimonious. So our formal definitions up to this point are not sensitive enough to capture the plausible intuition that Ψ^↑^ is both more inferentially modest and more informationally parsimonious than Ψ^→^.

One possible way of capturing this intuition is to use concepts from information theory, such as entropy. To do this, we must introduce a prior probability distribution Pr_0_ on the set ${\mathcal{H}}$ of all logically possible histories. In the example with the infinite grid, this could be the *uniform Bernoulli* distribution, which treats all the cells in the grid as independent, identically distributed random variables, where zero and one each appear with probability ½. Given two different symmetry monoids Ψ_1_ and Ψ_2_ that are consistent with the same nomological evidence *E*, we can use Pr_0_ to compare them: **Inferential modesty (relative to Pr**_**0**_**):** If Pr_0_(Ω(Ψ_2_, *E*)) ≤ Pr_0_(Ω(Ψ_1_, *E*)), then we say that Ψ_1_ is (at least weakly) more *inferentially modest* than Ψ_2_, relative to Pr_0_. **Informational parsimony (relative to Pr**_**0**_**):** Let *E*_1_ be the largest superset of *E* such that Ω(Ψ_1_, *E*_1_) = Ω(Ψ_1_, *E*). Let *E*_2_ be the largest superset of *E* such that Ω(Ψ_2_, *E*_2_) = Ω(Ψ_2_, *E*). If Pr_0_(*E*_2_) ≤ Pr_0_(*E*_1_), then we say that Ψ_1_ is (at least weakly) more *informationally parsimonious* than Ψ_2_, relative to Pr_0_.

Do these criteria enable us to prefer Ψ^↑^ to Ψ^→^, as intuition suggests? Let us begin with the second criterion. Comparing Figs. 4 and 5, we see that Pr_0_(*E*^→^) = 2^−5^, whereas Pr_0_(*E*^↑^) = 2^−3^, and so Ψ^↑^ is indeed more informationally parsimonious than Ψ^→^, relative to the uniform Bernoulli distribution. The first criterion, by contrast, does not help. Comparing Figs. 2 and 3, we see that Pr_0_(Ω(Ψ^→^, *E*)) and Pr_0_(Ω(Ψ^↑^, *E*)) are each zero, because they constrain an infinite number of cells. So, they do not differ in inferential modesty relative to Pr_0_. They do differ in more sensitive measures of inferential modesty, computed using more advanced notions from information theory, such as “entropy density”. But the details are beyond the scope of this paper.

Note that, if Ψ_1_ is more inferentially modest than Ψ_2_ in the original sense, which did not refer to any prior probability, then Ψ_1_ is more inferentially modest than Ψ_2_ in the information-theoretic sense, relative to *any* prior Pr_0_. This is because if Ω(Ψ_2_, *E*) ⊆ Ω(Ψ_1_, *E*), then Pr_0_(Ω(Ψ_2_, *E*)) ≤ Pr_0_(Ω(Ψ_1_, *E*)). Likewise, if Ψ_1_ is more informationally parsimonious than Ψ_2_ in the original sense, then Ψ_1_ is more informationally parsimonious than Ψ_2_ in the new sense, relative to *any* prior Pr_0_. The reason is that if *E*_2_ ⊆ *E*_1_, then Pr_0_(*E*_2_) ≤ Pr_0_(*E*_1_).

## Appendix D: Spatial distance in quantum mechanical systems

In Sect. 3.9, we proposed a definition of the *distance* between two regions *R* and *R′* in space, based on the minimum time required for a “signal” to travel from *R* to *R′*. As we have already observed, this definition is not entirely satisfactory in systems where signals can travel at arbitrarily high speeds (such as classical mechanics). This is particularly problematic in quantum mechanics, for two reasons.

First, there is the well-known phenomenon of *entanglement*, where two particles, perhaps separated by a large spatial distance, can apparently correlate their behaviour. But, in fact, this is less of a problem than it first appears. Rather than interpreting entanglement as “spooky action at a distance”, we can interpret it as a sign that we have not correctly specified the space *S* for this spatially extended system. A three-dimensional quantum system with *n* particles is not a collection of *n* wave functions on a three-dimensional space; rather, it should be viewed as a *single* wave function on a 3*n*-dimensional space. So, we should define *S* = **R**^3*n*^. Even if two particles appear widely “separated” from our three-dimensional perspective, their joint location is described by a single “hump” of the wave function in a six-dimensional space.^88^ From this perspective, the entangled behaviour of the two particles does *not* appear as a non-local phenomenon.

However, there is a more fundamental problem, which affects even a single-particle quantum system. Solutions to the Schrödinger equation on unbounded domains generally have *full support*: they give non-zero probability to every part of the space. This means, in effect, that the particle has a non-zero probability (albeit tiny) of “jumping” arbitrarily large distances through space.^89^ Thus, no two regions of space are ever unreachable from one another in any time duration, no matter how short, and so the distance between any two regions will be zero, according to the definition given in Sect. 3.9.

To address this problem, we must introduce a slightly more nuanced version of “unreachability”. Let ε > 0 be some small “error tolerance”. Given three events *E*, *F*, and *G* in Ω, we say that *E* and *G* are ε-*conditionally independent* *given* *F*, if $1-{\upvarepsilon } < \Pr _{F} (E \cap G){\text{/}}\Pr _{F} (E) \cdot \Pr _{F} (G) < 1 + {\upvarepsilon }$. In other words, the conditional probability Pr_*F*_(*E* ∩ *G*) is “almost” the same as the product Pr_*F*_(*E*)·Pr_*F*_(*G*), which means that *E* and *G* are “almost” conditionally independent, given *F*. If *R* and *R′* are two regions of *S*, and *t* > *t*_0_, then we say that *R′* is ε-*unreachable* from *R* in time *t* if, for any *h* in Ω, any event which happens in *R′* at time *t* is ε-conditionally independent of any event which happens in *R* at time *t*_0_, given the event $[h_{{R^{C}  \times \{ t_0\} }} ]$. If ε is small, this means that, *with very high probability*, a signal which originates in *R* at time *t*_0_ cannot reach *R′* before time *t*. We then define the ε-*distance* between *R* and *R′* to be the supremum of the set of all *t* such that *R′* is ε-unreachable from *R* in time *t* (if this supremum exists).

By using a small but non-zero ε, we can thus define a non-trivial notion of ε-distance between different regions of space, even in a quantum–mechanical system. This measure of distance will obviously depend on the value of ε, but it will roughly approximate the “classical” notion of distance. However, a detailed development of this approach is beyond the scope of this paper.

## Footnotes

1 Ideas from the theory of dynamical systems and stochastic processes have already begun to be applied to fields such as economics, biology, and cognitive science. See, e.g., van Gelder (1995), Auyang (1998), Juarrero (1999) and Silberstein and Chemero (2012).

2 We build on the formalism in List (2014) and List and Pivato (2015).

3 Conditional probability structures have previously been considered by several authors, e.g., Popper (1968), Rényi (1955), van Fraassen (1976), as reviewed in Halpern (2010).

4 Each *Pr*_*E*_ is defined on a suitable σ-algebra ${\mathcal {A}}$(Ω) on Ω; we set the technicalities aside. For any *E* ≠ ∅, *Pr*_*E*_ has the standard properties of a probability measure. But, for technical reasons, *Pr*_∅_(*D*) = 1 for all *D*.

5 For any subsets $C \subseteq D \subseteq E \subseteq \Omega$ we have $Pr_{E} (C) = Pr_{E} (D) \times Pr_{D} (C)$. Also, *Pr*_*D*_(*E*) = 1 for all $D \subseteq E \subseteq \Omega$.

6 A classical *dynamical system* consists of a set *X* (the *state space*) and a function ϕ from *X* into itself that determines how the state changes over time (the *dynamics*). Let *T* = {0, 1, 2, 3,…}. Given any state *x* in *X* (the *initial conditions*), the *orbit* of *x* is the history *h* defined by *h*(0) = *x*, *h*(1) = ϕ(*x*), *h*(2) = ϕ(ϕ(*x*)), and so on. Let Ω be the set of all orbits determined by (*X*, ϕ) in this way. Let {$Pr^{\prime}_{E}$}_*E*⊆*X*_ be any conditional probability structure on *X*. For any events *E* and *D* in Ω, we define *Pr*_*E*_(*D*) =  $Pr^{\prime} _{{E^{\prime}}} (D^{\prime})$, where *E′* and *D′* are the sets of all states in *X* whose orbits lie in *E* and *D*, respectively. Then {*Pr*_*E*_}_*E*⊆Ω_ is a conditional probability structure on Ω. Thus, Ω and {*Pr*_*E*_}_*E*⊆Ω_ together form a temporally evolving system. However, not every temporally evolving system arises in this way. While classical dynamical systems are deterministic, temporally evolving systems also subsume stochastic processes. Formally, a *stochastic process* is a temporally indexed collection of random variables {*X*_*t*_: *t* ∈ *T*} (with *T* as before) on some probability space (Ω, ${\mathcal {A}}$(Ω), *Pr*), where Ω is some underlying (abstract) set of possible worlds, ${\mathcal {A}}$(Ω) is a σ-algebra on Ω, and *Pr* is a probability measure on ${\mathcal {A}}$(Ω). For each time *t*, we can think of the random variable *X*_*t*_ as expressing the state of the stochastic process at *t*. To see that this gives rise to a temporally evolving system, note that each world ω in Ω induces a history *h* in our sense, where, for each *t*, *h*(*t*) is the realization of *X*_*t*_ at world ω. Most of the conditional probability structure {*Pr*_*E*_}_*E*⊆Ω_ can then be derived from *Pr* via Bayes rule, but {*Pr*_*E*_}_*E*⊆Ω_ may contain some additional information not encoded in *Pr* (namely, conditional probabilities arising from zero-probability events). In Sects. 3 and 4, we extend our framework to even more general classes of systems.

7 The use of such random forcings does not imply that certain features of the world are genuinely random. Instead, the “randomness” of such forcings is best understood *epistemically*—as a shortcut for an explicit and detailed description of the part of the world which lies outside the model. (This is true whether we adopt an *objectivist* or *subjectivist* interpretation of the probability structure overall.).

8 On these definitions, see also List (2014) and List and Pivato (2015). Related definitions of determinism (broadly, in terms of a history’s unique extendibility based on an initial state or segment) can be found, for instance, in van Fraassen (1989, section 10.4) and Butterfield (2012). See also the classic overview in Earman (1986) and the discussion of varieties of determinism in Sobel (1998). Earman shares our focus on local-to-global determination, as noted below. While Sobel discusses more than 90 variants of determinism, he frames the question differently than us. First of all, his focus, unlike ours in this paper, is on the free-will debate. Secondly, for us, the central question is (roughly): does the complete specification of the world (history) for a particular space–time region (e.g., a history’s initial segment) determine a complete specification of that world (history) for other space–time regions? By contrast, for Sobel, the central question is (roughly): does an event that occurs at a particular space–time location have a cause at some antecedent space–time location? Since we do not explicitly discuss the topic of causation here, Sobel’s analysis and ours are not immediately inter-translatable.

9 For discussion, see, e.g., Schaffer (2007) and List and Pivato (2015).

10 For related ideas, see also van Fraassen (1989, section 10.4).

11 We here employ, and subsequently generalize, a construction from List (2014).

12 Formally, we write ϕ^−1^(*E*) = {*h* in ${\mathcal {H}}$: ϕ(*h*) is in *E*}. The use of this notation for inverse images of sets does not imply that the function ϕ is invertible. Moreover, the inverse image of an event *E* could be empty, namely if none of the histories in *E* can be “reached” as transformations of other histories.

13 Strictly speaking, {*Pr*_*E*_}_*E*⊆Ω_ is only defined for subsets of Ω. But we can extend it as follows: for any *D*, *E* ⊆ H, if *E* ∩ Ω ≠ ∅, let ${Pr}_{E} (D) = {Pr}_{{E \cap \Omega }} (D \cap \Omega ).$Cases where *E* ∩ Ω = ∅ are not relevant here, because of the first bullet point in the definition of *symmetry*.

14 And further, there is a nomologically possible history, namely (*a*, *z*, *b*, *e*, *y*,…), such that applying the letter-shifting function to it yields the history (*b*, *a*, *c*, *f*, *z*,…).

15 Formally, *E′* consists of all the histories *h* in ${\mathcal {H}}$ such that ϕ(*h*) is in Ω and *h*(3) = *b.* Similarly, *D′* consists of all the histories *h* in ${\mathcal {H}}$ such that ϕ(*h*) is in Ω and *h*(5) = *z.*

16 Typically, we require ψ to be *order-preserving*, i.e., for all *t* and *t′* in *T*, if *t* *<* *t′*, then ψ(*t*) < ψ(*t′*). For example, if *T* = {1, 2, 3,…} with the standard ordering, the functions ψ(*t*) = *t* + 5 and ψ(*t*) = 5*t* are order-preserving. But we do not build this requirement into our definition of a time symmetry. Note that some time symmetries, such as time reversals in classical physical systems, are not order-preserving.

17 And further, there is some nomologically possible history *h″* = (*u*, *v*, *w*, *y*, *z*, *x*_1_, *x*_2_, *x*_3_,…) such that shifting the system’s state in *h″* five time periods into the future yields the history *h*. (Here, the exact values of *u*, *v*, *w*, *y*, *z* are irrelevant, as long as *h″* is nomologically possible.).

18 Note that classical dynamical systems have a particularly rich set of time symmetries. Let (*X*, ϕ) be a dynamical system, as defined in footnote 6. Suppose the function ϕ (which maps from *X* into itself) is *surjective*, i.e., for all *x* in *X*, there is some *y* in *X* such that ϕ(*y*) = *x*. Then the set Ω of orbits is invariant under all time-shifts. Let {*Pr′*_*E*_}_*E*⊆*X*_ be a conditional probability structure on *X*, and let {*Pr*_*E*_}_*E*⊆Ω_ be the conditional probability structure it induces on Ω. Suppose that {*Pr′*_*E*_}_*E*⊆*X*_ is ϕ-*invariant*, i.e., for any subsets *E* and *D* of *X*, if *E′* = ϕ^−1^(*E*) and *D′* = ϕ^−1^(*D*), then $Pr^{\prime} _{{E^{\prime}}} (D^{\prime}) = Pr^{\prime} _{{E}} (D)$. Then *every* time shift is a temporal symmetry of the resulting temporally evolving system. The study of dynamical systems equipped with invariant probability measures is the purview of *ergodic theory*.

19 As Roberts (2013) has argued, in general, a time reversal must not only map the time coordinate *t* to − *t*, but also apply an appropriate transformation to the system’s state at each point in time (in special cases, this could be the identity transformation, as in the case of a simple time reversal). Generally, we can think of the state of the system at each point in time as encoding not only some “static” properties (such as each particle’s position), but also some “kinetic” properties (such as each particle’s momentum). While static properties are preserved under time reversals, kinetic properties are not generally preserved. Similarly, in quantum mechanics, time reversals involve not only a reversal of the time coordinate, but also taking the conjugate of the wave function’s values.

20 To see that the set Ψ of *all* symmetries of a temporally evolving system forms a monoid, note that (i) the identity transformation is trivially a symmetry, and (ii) if two transformations each qualify as symmetries, by preserving the modal and probabilistic structure of the system, then so does their composition. If all symmetries are invertible, then Ψ becomes a *group*, but we need not assume this.

21 See, e.g., Wigner (1967), van Fraassen (1989, Part III), Mainzer (1996, section 5.3), Brading and Castellani (2003, 2013) and Baker (2010).

22 For example, Wigner (1967) recognizes the centrality of laws to scientific discovery, but does not define laws as facts that are invariant under symmetry. Instead, he seems to regard symmetry invariance principles as “second-order laws”, which relate laws to other laws (or which relate a law to itself). So, for him, it appears that laws establish relationships between events, and symmetries establish relationships between laws. Van Fraassen’s analysis (1989) is in many ways a precursor to ours (see, e.g., ibid., Section 11.2). But surprisingly he seems to reject the definition of laws as “facts invariant under symmetries” (ibid., Section 11.5). His proposed counterexamples (inspired by a passage from Weyl 1952) involve symmetry-invariant facts which are not nomologically necessary (such as the number of planets in the solar system). In contrast, we define laws as *nomologically necessary* facts which are invariant under symmetries. Mainzer (1996) alludes to the connection between symmetry and simplicity (esp. on p. 580), but does not offer a precise formal analysis. Brading and Castellani (2013) discuss the importance of symmetry in modern physics, but do not propose symmetry as a criterion for scientific law. Finally, Baker (2010) discusses the importance of symmetry arguments in metaphysics, especially in relation to the “identity of indiscernables”, but again does not propose symmetry as a criterion for scientific law. That said, our analysis clearly lies in the vicinity of what others have had in mind in their accounts of laws and symmetries.

23 Similarly, one can define the laws of *conservation of momentum*, *of angular momentum*, and so on.

24 One might raise the following concern. If *P* is nomologically necessary, and *h* is a possible history, then *h* satisfies not only *P*, but also all inverse images of *P* under all symmetries. To put it more simply, if *P* is nomologically necessary, then all its inverse images under all symmetries are *also* nomologically necessary. So, whenever *P* is nomologically necessary, we should be able to corroborate this fact via repeated testing, even if *P* is merely a brute necessity, rather than a symmetry-invariant law. At first sight, this may seem to challenge our claim that laws stand out in their testability. However, what have we really corroborated via the present exercise of repeated testing is not property *P* itself, but the much stronger (symmetry-invariant) property *P** defined above. And that property *P** is not merely nomologically necessary, but a law.

25 In a system without symmetries (aside from the identity transformation, which is trivially a symmetry), the distinction between laws and brute necessities could not be drawn. Every nomologically necessary property would then vacuously qualify as a “law”: it could not fail to be invariant under any symmetries. But this is clearly a degenerate case. As just argued, in such a system our ability to perform science would be seriously limited. Terminologically, one might distinguish between *trivial* and *non-trivial* laws. *Trivial laws* are ones that are vacuously symmetry-invariant (because there are no non-trivial symmetries). Our interest is of course in *non-trivial laws*.

26 Finally, it is worth emphasizing that, in our analysis, laws are always defined relative to a given system. When some property qualifies as a law of the system given by Ω and {*Pr*_*E*_}_*E*⊆Ω_, this does not imply that it will also qualify as a law of a different system, say Ω*′* and $\{ Pr^{{\prime }} _{{E^{{\prime }} }} \} _{{E^{{\prime }}  \subseteq \Omega ^{{\prime }} }}$. Different systems may be governed by different laws. Indeed, it is widely held that while our universe has certain laws of nature, other universes with distinct laws are logically possible. One might wonder whether our analysis only captures a notion of “system laws” rather than “laws of nature”. However, if we take the system given by Ω and {*Pr*_*E*_}_*E*⊆Ω_ to represent the universe as a whole, then our account can be interpreted as an account of the laws of nature. Further, as implied by “Appendix A”, the laws of a larger system, such as the universe, will constrain the laws of any subsystem that can be derived from it as a “factor system” (via constraining its symmetries). Thus, the laws of the universe will constrain the laws of any smaller subsystems in it. In Sect. 4.4, we show that structurally equivalent systems may share the same laws.

27 Mathematically, the pair (Ω, Ψ) can be interpreted as a classical *dynamical system*, as defined in footnote 6, with Ω playing the role of a state space (from an outside observer’s perspective) and the transformations in Ψ playing the role of state transformation rules.

28 Note that, under this heuristic interpretation, the world “forgets” its past history: from the perspective of Tuesday, it is as if Monday never happened. This is just an artefact of the formal mathematical model we are using in this example and has no deeper significance. If we used the set **Z** of all integers instead of the natural numbers to model time, it would obviate this issue.

29 See, e.g., Petersen (1989, Section 2.4), Walters (2000, Section 1.5, Definition 1.4), or Krengel (1985, Section 1.1.3, Definition 1.7) for precise definitions and further discussion. Ergodicity is usually defined for a symmetry monoid generated by a single transformation ψ, in which case the pair (*X*, ψ) is called an *ergodic* *dynamical system*. But the definition generalizes immediately to arbitrary symmetry monoids. See, e.g., Krengel (1985, p. 203). At first sight, this seems unrelated to the use of the term in probability theory, where a stochastic process (such as a Markov process or random field) is called *ergodic* if it has a unique stationary measure. But the two definitions are related because any stochastic process can be represented as a dynamical system (Petersen 1989, Sections 1.2B to 1.2D). Finally, in statistical physics, Boltzmann’s Ergodic Hypothesis conjectures that the fraction of the time that a physical system spends in a particular region of its state space is proportional to the size of that region. This was the inspiration for the Birkhoff Ergodic Theorem (see below).

30 If Ω is infinite, there is a subtle distinction between *almost no* *history* (*Pr*_Ω_(*E*) = 0) and *no history* (*E* = ∅). Likewise, *almost every* *history* (*Pr*_Ω_(*E*) = 1) is subtly distinct from *every history* (*E* = Ω).

31 This insight is the basis for Reichenbach’s (1949) “straight rule”, which is to take observed frequencies as the best estimates of “true” probabilities. See, e.g., Eberhardt and Glymour (2009).

32 This result is often called the *Birkhoff (or Pointwise) Ergodic Theorem*. For simplicity, we have stated it relatively informally. For formal statements, see Krengel (1985, Section 1.2), Petersen (1989, Section 2.2), Walters (2000, Theorem 1.14), or Berkovitz et al. (2006). Further, we have defined ergodicity for a discrete set of time symmetries. More generally, the statements in this section hold for any set of symmetries that forms an *amenable monoid*. See Krengel (1985, Section 6.4).

33 The literature contains many proposals on how to formalize Occam’s Razor. See, e.g., Baker (2013) and Fitzpatrick (2015). For an efficiency argument for Occam’s Razor, see Kelly (2007).

34 Mainzer (1996, Section 5.3, p. 580) also relates symmetry to simplicity and notes that scientists generally prefer simpler theories. Likewise, van Fraassen (1989, Section 10.2) notes that when constructing a scientific model, we generally assume that the model satisfies a given symmetry unless we have good reason to believe the contrary. His slogan is: “an asymmetry must always come from an asymmetry”, with some caveats. But neither of these authors connects these ideas to Occam’s Razor.

35 In the present terms, rotations must be represented as transformations of the state space *X*. In Sect. 3.5, we represent rotations more explicitly, relying on a formal representation of space.

36 The importance of Markov properties in understanding causality has been emphasized by Pearl (2000) and Spirtes et al. (2000).

37 If *Pr*_Ω_(*D*) > 0, the first equation is equivalent to *Pr*_Ω_(*E* ∩ *D*) = *Pr*_Ω_(*D*) *Pr*_Ω_(*E*). If *Pr*_Ω_(*E*) > 0, the second equation is equivalent to *Pr*_Ω_(*E* ∩ *D*) = *Pr*_Ω_(*D*) *Pr*_Ω_(*E*). Thus, if *Pr*_Ω_(*D*) > 0 and *Pr*_Ω_(*E*) > 0, the two equations are equivalent. But if *Pr*_Ω_(*D*) = 0 or *Pr*_Ω_(*E*) = 0, the equations must be stated separately.

38 Technically, the system just described is a *simple random walk*.

39 Here, to avoid cumbersome subscripts, we are using the notation *Pr*(*A* | *B*) to denote the conditional probability *Pr*_*B*_(*A*).

40 We are *not* saying that this is the most parsimonious or computationally convenient way to represent a classical mechanical system. It is only one way of representing such a system in our framework.

41 Strictly speaking, particles in quantum systems do not have “positions”, so we are using this term rather loosely. Also, there is a dual representation of the wave function (obtained via Fourier transform), where the coordinates in (**R**^3^)^*n*^ represent the “momenta” (again, loosely) of the *n* particles. These two representations are equally valid.

42 For an earlier discussion of local-to-global forms of determinism, see Earman (1986, pp. 33–35).

43 This sort of determinism occurs in *expansive cellular automata*, a class of spatially extended systems discussed in the theory of dynamical systems. See Pivato (2009).

44 For example, this phenomenon arises frequently in the solution of *boundary value problems* in mathematical physics. See Pivato (2010).

45 For an example, take an *n*-particle quantum system, where *S* = (**R**^3^)^*n*^, *X* is the set of complex numbers, and a spatially extended history *h* is a wave function. Let ϕ be a *phase rotation map* on the complex plane; formally, there is some angle θ such that, for all *x* in *X*, ϕ(*x*) = e^*i*θ^*x*. Then ϕ is a state symmetry.

46 As before, for any subsets *D*, *E* of $\cal{H}$, we define *Pr*_*E*_(*D*) = *Pr*_*E*∩Ω_(*D* ∩ Ω), provided *E* ∩ Ω ≠ ∅.

47 An additional property we might require of a space–time transformation ψ is *time preservation*: for any points $(s_{1} ,t_{1} )$ and $(s_{2} ,t_{2} )$ in *S* × *T*, with $\uppsi(s_{1} ,t_{1} ) = (s_{1}^{\prime } ,t_{1}^{\prime } )$ and $\uppsi(s_{2} ,t_{2} ) = (s_{2}^{\prime } ,t_{2}^{\prime } )$, if $t_{1} \le t_{2}$, then $t_{1}^{\prime } \le t_{2}^{\prime }$. This implies that if $t_{1} = t_{2}$, then $t_{1}^{\prime } = t_{2}^{\prime }$. A time-preserving transformation acts on *S* × *T* such that the set of all space–time points at time *t*_1_ gets moved *en bloc* to the set of all space–time points at time $t^{\prime}_{1}$. The transformations described above have this property, but we do not need to include it in our definition.

48 These are defined as follows. *Spatial rotation*: Fix a line *L* in *S* and an angle θ. For any point *s* in *S*, let *s′* be the point obtained by rotating *s* by an angle of − θ around *L*. For all (*s*, *t*) in *S* × *T*, define $\uppsi(s,t) = (s^{{\prime }} ,t)$. Let *L′* be the line parallel to *L*, but passing through the origin. For all *x* in *X*, define ϕ(*x*) by rotating all the momentum vectors and force field vectors in *x* by the angle θ around *L′. Spatial reflection*: Fix a plane *P* in *S*. For any point *s* in *S*, let *s′* be the point obtained by reflecting *s* across *P*. For all (*s*, *t*) in *S* × *T*, define $\uppsi(s,t) = (s^{{\prime }} ,t)$. Let *P′* be the plane parallel to *P*, but passing through the origin. For all *x* in *X*, define ϕ(*x*) by reflecting all the momentum vectors and force field vectors in *x* across *P′*. *Spatial rescaling*: Fix some real number *r* > 0, and define $\uppsi(s,t) = (s /r,\;t)$ for all (*s*, *t*) in *S* × *T*. Meanwhile, let ϕ be a transformation of *X* that multiplies the momentum vector of every particle by *r*, and also multiplies all force field vectors by *r*. *Galilean transformation*: For all (*s*, *t*) in *S* × *T*, define $\uppsi(s,t) = (s - tv,t)$, where *v* is a fixed three-dimensional vector (an element of **R**^3^). Meanwhile, for all *x* in *X*, define ϕ(*x*) by adding the vector *v* to all momentum vectors in *x*.

49 In all four examples in footnote 48, neither ψ nor ϕ is itself a symmetry. But when combined, they *do* form a symmetry. For another example, in classical electrodynamics, let ψ be a spatial reflection acting on $S \times T$, and let ϕ be a transformation of *X* which applies the corresponding reflection to all momentum vectors and field vectors, and which further *negates* the magnetic field vector. Neither one of these transformations is a symmetry by itself, but when combined, they *do* form a symmetry.

50 The system might also have other spatiotemporal symmetries, but this is irrelevant here.

51 For simplicity, we here assume that the symmetry monoid Ψ is isomorphic to **Z**^4^, where **Z** is the monoid of integers. The theorem, which we have stated somewhat informally, also holds if Ψ is isomorphic to **R**^4^, or if Ψ is any amenable monoid. For a more formal statement, see Krengel (1985, Chapter 6).

52 It is not necessary to average over a sequence of “boxes”; the same argument works for any sequence of sets which increase in size and thickness in an appropriate sense, technically any *Følner sequence*.

53 One complication is that not all systems are ergodic. For example, in systems with conservation laws, such as conservation of energy or momentum, each value of the conserved variables determines a non-trivial invariant subset of Ω. But the “Ergodic Decomposition Theorem” shows that any non-ergodic system can be split up into “ergodic components”—informally, minimal invariant subsets of Ω, each of which (except possibly a set of measure zero) supports its own ergodic probability function (see, e.g., Glasner 2003, p. 72, Theorem 3.22). If we are part of the system, then we are already confined to one such component. Furthermore, even if the system as a whole is not ergodic, many of its factor systems may be ergodic (see “Appendix A”). This suggests that, by choosing the right level of description for the system (e.g., by adopting a sufficiently coarse-grained, higher-level description, as discussed in List and Pivato 2015), we may be able to reap the benefits of ergodicity. For the applicability of ergodic methods to non-ergodic Hamiltonian systems, see Berkovitz et al. (2006, Section 4).

54 Adjacency structures are similar to proximity relations, which have been studied extensively in general topology (e.g., Willard 1970, Sections 40 and 41). But we do not assume that our adjacency structures satisfy the axioms of a proximity relation, so they are more versatile.

55 Generally, an adjacency structure can be defined in a similar way on any metric or topological space.

56 Generally, an adjacency structure can be defined in a similar way on any Cayley graph of any group.

57 See Ilachinski (2001) and Moore and Mertens (2011).

58 Formally, (i) the operation + is *associative*, i.e., (*t*_1_ + *t*_2_) + *t*_3_ = *t*_1_ + (*t*_2_ + *t*_3_) for all *t*_1_, *t*_2_, *t*_3_ in *T*; (ii) there is an *identity* element, namely *t*_0_, such that *t*_0_ + *t* = *t* = *t* + *t*_0_ for all *t* in *T*; (iii) every element *t* in *T* has an *inverse* − *t* such that *t* + (− *t*) = *t*_0_ = (− *t*) + *t*; and (iv) the ordering < is *left-homogeneous*, meaning that, for all *t*_1_, *t*_2_, *t*_3_ in *T*, we have (*t*_1_ + *t*_2_ < *t*_1_ + *t*_3_) ⇔ (*t*_2_ < *t*_3_). Left-linearly ordered groups are not generally *commutative* (“*abelian*”), i.e., we could have *t*_1_ + *t*_2_ ≠ *t*_2_ + *t*_1_ for some *t*_1_, *t*_2_ in *T.* See, e.g., Fuchs (2011) for an introduction to ordered groups.

59 In our definition of “non-reachability”, we have referred to the reference time *t*_0_. However, because Ψ acts freely and transitively on *T*, the reference time does not matter. When region *R′* is not reachable from region *R* in time *t*, this implies that, for *any* times *t*_1_ and *t*_2_ with *t*_2_ − *t*_1_ = *t*, any event which happens in *R′* at time *t*_2_ is conditionally independent of any event which happens in *R* at *t*_1_, given $[h_{{R^{C}  \times \{ t_1\} }} ]$.

60 If the maximum does not exist, we can use the supremum, provided the order of time is *supremum-complete* (i.e., any subset of *T* has a supremum), as it is if *T* is the set of real numbers. If the order of time is not supremum-complete, then the precise distance between *R* and *R′* may not be well-defined.

61 Technically, this means that there exist events *E*_1_, *E*_2_, and *E*_3_ in Ω which happen, respectively, in region *R*_1_ at time *t*_1_, in region *R*_2_ at time *t*_1_, and in region *R*_3_ at time *t*_2_ such that *E*_1_, *E*_2_, and *E*_3_ are *pairwise independent*, but not *jointly independent*. This situation is common in probability theory.

62 To be precise, $d(R,R^{{\prime }} ) = \min \{ d(s,s^{{\prime }} ):s \in R\;{\text{and}}\;s^{{\prime }}  \in R^{{\prime }} \}$. Strictly speaking, this only works if *R* and *R′* are *finite* sets of points. For *infinite* sets, we would need a slightly stronger version of non-complementarity, which says that $d(R,R^{{\prime }} ) = {\text{inf}} \{ d(s,s^{{\prime }} ):s \in R\;{\text{and}}\;s^{{\prime }}  \in R^{{\prime }} \}$ (and this infimum exists).

63 Note that, in the literature on general relativity theory, the word “event” is used to refer to the objects we call “loci”. Our use of the word “event” is consistent with its use in probability theory.

64 In a model of general relativity, a similar construction works if *R′* is a Cauchy surface in the four-dimensional space–time manifold.

65 A subset *R* of *I* is *open* if, for any *s* in *R*, there is some *r* > 0 such that the ball of radius *r* around *s* is contained in *R*.

66 We are not the first to suggest that the geometry and/or topology of space–time could be an emergent property of more fundamental causal structures. Brown and Pooley (2001, 2006) have argued that the geometry of relativistic space–time should be seen as a consequence of the symmetries (i.e., Lorentz covariance) of the dynamical laws governing matter and electromagnetism. In their words (2006, Section 5): “space–time’s Minkowskian structure cannot be taken to explain the Lorentz covariance of the dynamical laws. From our perspective, of course, the direction of explanation goes the other way around. It is the Lorentz covariance of the laws that underwrites the fact that the geometry of space–time is Minkowskian.” See also Brown (2005). However, Brown and Pooley’s approach is very different from ours. The idea of emergent space–time geometry also appears in the literature on high-energy physics and quantum cosmology. See, e.g., Konopka et al. (2008) and Hamma et al. (2010).

67 To be more precise: given a possible time structure on *I*, we can represent the amorphous system as a temporally evolving system, and this system, in turn, will satisfy the temporal Markov property. The construction is straightforward, but to avoid too many technicalities, we set aside the details here.

68 This is *not* the same as thermodynamic entropy, although it is loosely related. Thus, the discussion that follows should *not* be interpreted thermodynamically.

69 If |*X*| and $|R^{{\prime }}|$ are the cardinalities of *X* and *R′*, then the cardinality of $\Omega_{R^{{\prime }}}$ is at most $|X|^{{|R^{{\prime }} |}}$.

70 Formally, ${{\upeta}} ({R}^{\prime},  h_{R})$ is the sum, over all possible *R′*-restrictions $h_{R^{{\prime }}}$ in $\Omega_{R^{{\prime }}}$, of

$$ - Pr([h_{{R^{{\prime }} }} ]|[h_{R} ])\log_{2} [Pr([h_{{R^{{\prime }} }} ]|[h_{R} ])]/|R^{{\prime }} |\log_{2} (|X|). $$

However, the precise formula is not important for this discussion.

71 To be more precise, it is the *supremum* of this set. The maximum is not always well-defined.

72 Even if η(τ, *t*) = 0, there may be some “residual” unpredictability, in that Ω may contain more than one extension of *h*_*R*_ to all of *I*. However, the conditional probability structure {*Pr*_*E*_}_*E*⊆Ω_ concentrates all probability on *one* of these possible extensions; the rest of the extensions get probability zero.

73 Again, strictly speaking, we require the supremum of η(τ, *t*) across all *t*. This is not the only possible measure of the system’s unpredictability under time structure τ. We could also take the average or some other aggregate measure. For example, suppose that *I* is an *N-*dimensional integer lattice (i.e., *I* = **Z**^*N*^). Then we could measure the system’s unpredictability under different time structures using the theory of *entropy geometry* and *expansive subdynamics* first developed for multidimensional cellular automata by Milnor (1988) and later extended to arbitrary multidimensional symbolic dynamical systems by Boyle and Lind (1997). See the section on “Entropy” in Pivato (2009) for a summary.

74 On a broadly “structuralist” or “relational” approach to metaphysics, see, e.g., Ladyman and Ross (2009). On “absolute” versus “relational” accounts of space and time, see, e.g., Earman (1989). On “substantivalism” and its discontents, see, e.g., Nerlich (2003).

75 In fact, any bijective symmetry of a system constitutes an isomorphism from a system into itself.

76 This construction, under the present notational conventions, was introduced in List (2014) and List and Pivato (2015). Relatedly, see also Yoshimi (2012).

77 For earlier work defending higher-level chance, sometimes using a strategy similar in spirit to the present one (though not fully equivalent), see, e.g., Loewer (2001), Frigg and Hoefer (2010), Glynn (2010), Strevens (2011) and Hemmo and Shenker (2012).
